# Ameliorative impacts of polymeric and metallic nanoparticles on cisplatin-induced nephrotoxicity: a 2011–2022 review

**DOI:** 10.1186/s12951-022-01718-w

**Published:** 2022-12-01

**Authors:** Maryam Davoudi, Yasaman Jadidi, Kiana Moayedi, Vida Farrokhi, Reza Afrisham

**Affiliations:** 1 Department of Clinical Laboratory Sciences, Faculty of Allied Medicine, Tehran University of Medical Sciences, Tehran, Iran; 2 Department of Clinical Biochemistry, Faculty of Medicine, Tehran University of Medical Sciences, Tehran, Iran; 3 Department of Hematology, Faculty of Allied Medicine, Tehran University of Medical Sciences, Tehran, Iran

**Keywords:** Cisplatin, Nephrotoxicity, Metallic nanoparticles, Polymers, Antioxidant, Anti-apoptosis, Nephroprotection, Kidney

## Abstract

Cisplatin (CDDP) is a well-known platinum-based drug used in the treatment of various malignancies. However, the widespread side effects that this drug leaves on normal tissues make its use limited. Since cisplatin is mainly eliminated from the kidneys, CDDP-induced nephrotoxicity is the most significant dose-limiting complication attributed to cisplatin, which often leads to dose withdrawal. Considering the high efficiency of cisplatin in chemotherapy, finding renoprotective drug delivery systems for this drug is a necessity. In this regard, we can take advantages of different nanoparticle-based approaches to deliver cisplatin into tumors either using passive targeting or using specific receptors. In an effort to find more effective cisplatin-based nano-drugs with less nephrotoxic effect, the current 2011–2022 review study was conducted to investigate some of the nanotechnology-based methods that have successfully been able to mitigate CDDP-induced nephrotoxicity. Accordingly, although cisplatin can cause renal failures through inducing mitochondria dysfunction, oxidative stress, lipid peroxidation and endoplasmic reticulum stress, some CDDP-based nano-carriers have been able to reverse a wide range of these advert effects. Based on the obtained results, it was found that the use of different metallic and polymeric nanoparticles can help renal cells to strengthen their antioxidant systems and stay alive through reducing CDDP-induced ROS generation, inhibiting apoptosis-related pathways and maintaining the integrity of the mitochondrial membrane. For example, nanocurcumin could inhibit oxidative stress and acting as a ROS scavenger. CONPs could reduce lipid peroxidation and pro-inflammatory cytokines. CDDP-loaded silver nanoparticles (AgNPs) could inhibit mitochondria-mediated apoptosis. In addition, tea polyphenol-functionalized SeNPs (Se@TE) NPs could mitigate the increased level of dephosphorylated AKT, phosphorylated p38 MAPK and phosphorylated c-Jun N-terminal kinase (JNK) induced by cisplatin. Moreover, exosomes mitigated cisplatin-induced renal damage through inhibiting Bcl2 and increasing Bim, Bid, Bax, cleaved caspase-9, and cleaved caspase-3. Hence, nanoparticle-based techniques are promising drug delivery systems for cisplatin so that some of them, such as lipoplatins and nanocurcumins, have even reached phases 1–3 trials.

## Introduction

Cisplatin (cis-diamminedichloroplatinum (CDDP)) is a platinum (Pt)-based chemotherapeutic compound with the chemical formula of cis-[PtCl_2_(NH_3_)_2_], which is widely used to treat solid tumors [1]. Cisplatin can enter different cells via both passive transport and facilitated diffusion (2). Due to the high concentration of chloride ions in the extracellular space, CDDP is not able to be hydrolyzed in the blood. However, upon the cell entry, the low concentration of intracellular chloride ions causes the gradually hydrolysis of CDDP to its highly reactive forms, i.e. [Pt(NH_3_)_2_Cl(OH_2_)]^+^ and [Pt(NH_3_)_2_(OH_2_)_2_]^2+^ [2–5]. These cationic derivatives can easily react with intracellular nucleophiles such as sulfur-containing proteins (e.g., glutathione (GSH)), histones, DNAs and RNAs [2, 5, 6].

The main anticancer mechanism proposed for CDDP is the induction of DNA damage through forming cross-linking complexes with purine residues [2, 5, 6]. If DNA repair processes cannot be completed, the cell will undergo apoptosis. Furthermore, GSH is also conjugated to CDDP and prevents it from binding to DNA, thus resulting in cisplatin resistance [4]. In addition to drug resistance, the non-selective drug delivery of cisplatin makes its use challenging because it has cytotoxic effects on normal cells as well as cancerous cells. Consequently, cisplatin shows cytotoxic impacts on kidneys, ears, neurons, heart, liver, gastrointestinal tract, and blood cells [7, 8].

Since CDDP is excreted predominately in the urine, it accumulates more in kidneys than other organs. Therefore, nephrotoxicity is the most important side effect attributed to CDDP [9]. Cisplatin can be directly absorbed and concentrated in the proximal tubules of the kidney and induce CDDP-dependent apoptosis and necrosis [10]. Hence, cisplatin cause kidney injury through inducing mitochondria vacuolation, oxidative stress, lipid peroxidation and endoplasmic reticulum (ER) stress [11, 12]. These nephrotoxic effects induce electrolyte imbalances, hematuria, proteinuria, reduced glomerular filtration rate, and high serum levels of creatinine and urea [9, 12, 13]. Therefore, understanding the mechanisms of cisplatin-induced nephrotoxicity and finding effective approaches to manage them is a necessity in the field of chemotherapy.

The use of cisplatin derivatives, i.e., carboplatin [di-amine(1,1-cyclobutadicarboxylato)Pt (II)] and oxaliplatin [(1R,2R)-diaminocyclohexane)oxalate-Pt(II)], is one of the most effective methods to mitigate cisplatin-induced nephrotoxicity. Instead of the chlorides in the chemical formula of cisplatin, these drugs have cyclobutane-1, 1-dicarboxylato and oxalate, which can chelate Pt more strongly [14]. Thus, the chelating ligands can be substituted by water far more slowly [15]. As a result, they mitigate CDDP-induced systemic toxicity. However, these agents also exhibit less efficacy or antitumor activities than cisplatin [16–18]. Moreover, the main drawbacks of chemotherapeutic drugs are usually related to their solubility, biodistribution and ability to enter cells. Therefore, finding other approaches is crucial to resolve all of these issues [19].

**Fig. 1:**
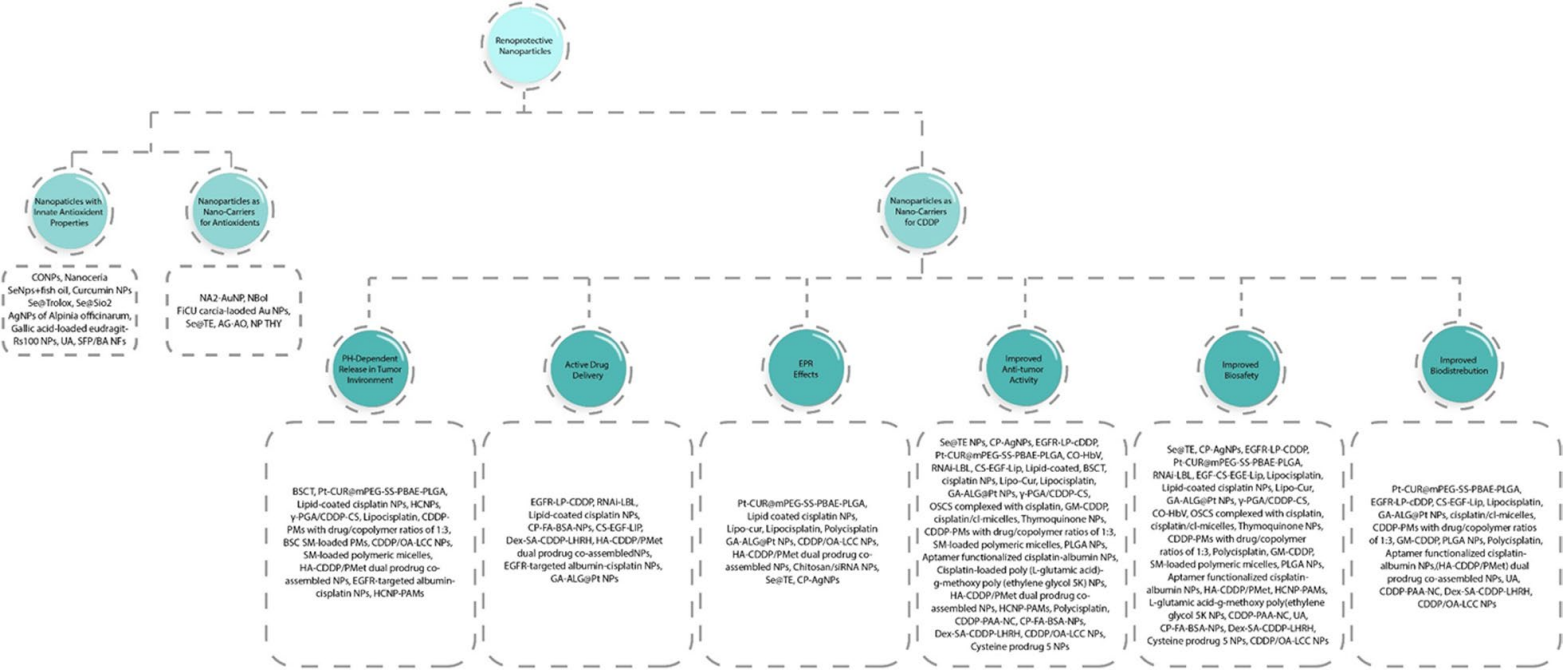
Renoprotective nanoparticles classification.Cerium oxide nanoparticles (CONPs); Selenium nanoparticles (SeNPs); 6-hydroxy-2,5,7,8-tetramethylchroman-2-carboxylic acid (trolox) surface-functionalized selenium nanoparticles (Se@Trolox); urolithin A (UA); Silk fibroin peptide/baicalein nanofibers (SFP/BA NFs); N-(2-hydroxyphenyl) acetamide-conjugated gold nanoparticles (NA2-AuNPs); PLGA-encapsulated nano-Boldine (NBol); tea polyphenol-functionalized SeNPs (Se@TE NPs); Thymoquinone nanoparticles (NP THY); N-benzylN,O-succinyl chitosan (BSCT); The platinum complexes of curcumin (Pt-CUR@ mPEG-SS-PBAE-PLGA); Hyaluronan–cisplatin conjugate nanoparticles (HCNPs); CDDP complexed with γ-polyglutamic acid and chitosan (γ-PGA/CDDP-CS); Cisplatin-loaded polymeric micelles (CDDP-PMs) (with drug/copolymer ratios of 1:3); Silymarin-loaded benzyl-functionalized succinyl chitosan (BSC (SM-loaded PMs); Hyaluronic acid cisplatin/polystyrene-polymetformin (HA-CDDP/PMet); Epidermal growth factor receptor (EGFR); Pt (IV) prodrug-loaded ligand-induced self-assembled nanoparticles (GA-ALG@Pt NPs); CaCO3 nanoparticle (CDDP/OA-LCC NPs); Cisplatin-loaded green silver nanoparticles (CP-AgNPs); Carbon monoxide(CO)-loaded hemoglobin-vesicle (CO-HbV); RNAi-Chemotherapy Layer by Layer Nanoparticle (RNAi-LBL); Cisplatin-sodium alginate conjugate liposomes modified with EGF (CS-EGF-Lip); Chitosan derivatives, N-octyl-N,O-succinyl chitosan (OSCS); Gelatin microspheres incorporating cisplatin (GM-CDDP); Hyaluronan–cisplatin conjugate nanoparticles (HCNPs) entrapped in Eudragit S100-coated pectinate/alginate microbeads (PAMs) (HCNP-PAMs); Cisplatin-polyacrylic acid (PAA) nano capsule (CDDP-PAA-NC); Cisplatin loaded folic acid decorated bovine serum albumin nanoparticles (Cp-FA-BSA-Nps); Cisplatin-loaded LHRH-modified dextran nanoparticles (Dex-SA-CDDP-LHRH)

Nanotechnology is a cutting-edge approach that may be effective in developing some drug delivery systems for CDDP. As shown in Fig. 1, and Tables 1 and 2, many studies have already taken advantages of nanocarriers to improve the efficacy of CDDP and reduce their nephrotoxicity [20]. In this regard, the kidneys are responsible for filtering and removing molecules smaller than 6 nm from blood. Therefore, the size of nanoparticles is a crucial factor to prevent the drug from entering the kidney [21, 22]. Moreover, NPs can passively accumulate in cancerous tissue owing to the enhanced permeability and retention (EPR) effect, which allows nanometer-sized carriers to be accumulated more in tumors than normal tissues [23]. Additionally, more than 90% of circulating cisplatin can be inactivated through irreversibly attaching to albumin [24]; therefore, the proper design of nanocarriers can also prevent the cisplatin from inactivation. Furthermore, these nano-drugs can selectively deliver higher concentrations of CDDP to tumor, which prevents the drug from distributing in other organs and inducing the dose-limiting cytotoxic effects on normal cells. Finally, many studies had already shown that a large portion of cisplatin (about 27–50%) was excreted by the kidneys within 48 h of injection, so the use of nano-carriers may also be effective in increasing the bioavailability of cisplatin [25].

**Table 1:** The renoprotective mechanisms of different metallic and polymeric nanoparticles

Nanoparticles	Size (nm)	Zeta potential (mV)	Treatment	Renoprotective mechanisms	Ameliorative effects on kidneys	Refs.
Metallic nanoparticles						
Cerium oxide nanoparticles (CONPs)	Less than 25	–	Albino rats (60 mg/kg) (i.p.)	-As an anti-oxidant and anti-inflammatory agent	-Decreased: urea, creatinine, and histopathological damages -Increased: IL-10 and TAO	[52]
Nanoceria	113	−15.4	Swiss mice (0.2 and 2 mg/kg) (i.p.)	-Anti-oxidant and anti-inflammatory activities	-Decreased: urea, creatinine, lipid peroxidation, pro-inflammatory cytokines, and histopathological damages -Increased: catalase and GSH	[53]
Green silver nanoparticles (CP-AgNPs)	33.2	–	Wistar rats (2.5 mg/kg) (i.p.)	-Inhibiting mitochondria-mediated apoptosis	- Decreased: Bax, caspase-3, and histopathological damages -Increased: Bcl-2 -Inhibited: the releases of AIF and cytochrome c from mitochondria	[54]
N-(2-hydroxyphenyl) acetamide-conjugated gold nanoparticles (NA2-AuNPs)	25–60	−45.7	Balb/c mice (Different doses) (i.p.)	-As a nano-carrier for a natural anti-oxidant	-Downregulated: NF-κB, iNOS and IL-6 -Upregulated: HO-1	[55]
Ficus carica L. leaves extract-loaded AuNPs	100	–	Albino rats (0.5 ml of Au NPs/Fig mixture as different v/v ratios) (orally)	-As a nano-carrier for a natural anti-oxidant	-Scavenging ROS -Mitigating AKI severity	[56]
Selenium nanoparticles (SeNPs)	45.9	–	Pretreatment of albino rats with both SeNPs (0.5 mg/kg; orally) and fish oil before cisplatin administration and γ-radiation	-Inhibiting caspase-dependent apoptosis and inflammation -Strengthening the antioxidant system	-Decreased: urea, creatinine, TNF-α, caspase-3, cyclooxygenase-2, and histopathological damages	[57]
Tea polyphenol (TP)-functionalized SeNPs (Se@TE)	Under microwave conditions: -After 0.5 h: 200.8 -After 1 h: 216.4 -After 2 h: 220.4	-After 0.5 h: − 9.4 -After 1 h: − 11.5 -After 2 h: − 10.7	-In vitro: HK-2 cells -In vivo: KM mice (1 and 2 mg/kg) (i.v.)	-As a nano-carrier for a natural anti-oxidant -Prevention of mitochondrial dysfunction -Activating seleno-enzymes	-Inhibited: dephosphorylation of AKT, phosphorylation of p38 MAPK, phosphorylation of JNK, phosphorylation of p53, pro­apoptotic genes (i.e., Bax and Bad), caspase-mediated apoptosis, and ROS production -Upregulated: anti-apoptotic genes (i.e., Bcl2 and Bcl-xL)	[58]
6-hydroxy-2,5,7,8-tetramethylchroman-2-carboxylic acid (trolox) surface-functionalized SeNPs (Se@Trolox)	100	Lower than -50	-In vitro: HK-2 cells	-Inhibiting apoptosis	-Blocked: ROS-induced p53 phosphorylation -Regulated: AKT/MAPK pathways	[59]
Se@SiO2 nanocomposites	53	–	-In vitro: HK-2 cells -In vivo: pretreatment of C57BL/6 mice with Se@SiO2 (200 μl, 2 mg/ml) (i.v.)	-Reversing cisplatin-induced tubular damage	-Decreased: TNF­α and IL-6 -Activated: Sirt1 at both in-vivo and in-vitro	[60]
A. officinarum-silver nanoparticles (AG-AO)	100	–	Wistar rats (50 mg/kg/day or/and 100 mg/kg/day) (orally)	-As a nano-carrier for a natural anti-oxidant	-Decreased: ROS levels, Bax, p53, caspase­3 and ­9 proteins, TNF­α, IL­1β, NF-κB and NO pathways -Increased: Bcl2 protein, SOD, CAT and GSH -Inhibited: tubular atrophy, interstitial edema, necrosis and inflammation	[62]
Polymeric nanoparticles						
Nanocurcumin (NC)	240.7	–	Sprague–Dawley rats (100 mg/kg/day) (orally)	-Changing the expression of cisplatin transporters on renal cells	-Increased: OCT2	[66]
Curcumin nanoparticles (CUR NPs)	10–50	–	Wistar albino rats (50 mg/kg body wt/day) (orally)	-Maintaining the mitochondrial function	-Decreased: lipid peroxidation, NO and TNF-α Increased: GSH and Na^+^/Ka + -ATPase activity	[73]
The platinum complexes of curcumin (Pt-CUR@ mPEG-SS-PBAE-PLGA)	154.87	−20.8	In vitro: HEK-293 cells In vivo: Balb/c nude mice (1 mg/kg of Pt-CUR) (tail intravenous injection)	-Redox and pH-dependent release	-Suppressed: ROS production	[74]
Curcumin nanoparticles (CURNPs)	2–100	–	Rats (30 or 60 mg/kg b.w) (orally)	-As an antioxidant	-Decreased: urea, creatinine, F-2IsoPs, and histopathological damages -Increased: SOD and CAT activities	[72]
SinaCurcumin^®^	–	–	Patients with localized muscle-invasive bladder cancer (180 mg/day)	-A complementary therapy	-No significant difference between nanocurcumin group and placebo group in response to grade 3/4 renal	[76]
Cisplatin-incorporated liposome conjugated with EGFR antibodies (EGFR:lP-cDDP)	247.9		Balb/c nude mice (10 mg/kg) (i.v.)	-Targeted cisplatin delivery	-No pathological changes	[78]
Cisplatin-sodium alginate conjugate liposomes modified with EGF (CS-EGF-Lip)	112.37	−23.83	Balb/c nude mice (i.v.)	-Targeted cisplatin delivery	-Decreased: urea and creatinine	[79]
RNAi-Chemotherapy Layer by Layer Nanoparticle	180	−30	Mice (i.v.)	-RNA-based therapeutics	-Increased: urea and creatinine at nanoparticles contained 16 mg/kg of cisplatin -Decreased: cisplatin-induced nephrotoxicity	[80]
Lipoplatin	–	–	Patients with both cancer and renal failure	-Monotherapy -co-therapy with 5-fluorouracil-leucovorin or gemcitabine or paclitaxel	Creatinine at a normal level	[81]
Lipocisplatin	104.4	–	Balb/c mice (6.5 mg Pt/kg) (tail intravenous injection)	-The size of cisplatin was more than cutoff for renal clearance	-Less drugs in kidneys	[82]
Curcumin-loaded liposomes synthesized by microfluidic technology (Lipo-Cur)	120	–	Balb/c mice (20 mg/kg) (i.v. and orally)	–	-Decreased: urea, creatinine, and histopathological damages after a single dose	[83]
Nanoparticulated honokiol	–	–	(ICR mice 5 mg/kg b.m) (Tail-vein injection)	-As a nano-carrier for a natural anti-oxidant -Inhibiting inflammation and fibrosis	-Inhibited: caspase-3-mediated apoptosis -Reversing acute kidney injury	[84]
Lipid-coated cisplatin nanoparticles	63.6	+ 12.8	Xenograft mice	-Microneedle mediated delivery -pH-dependent release	-Decreased: urea, creatinine, and histopathological damages	[85]
Cisplatin NanoComposite (CHIT/Cis/MgO NPs)	–	–	Wister rats (5.75 mg/kg b. wt.day) (i.p.)	-Sustained release	-Compared to cisplatin, it induced less malondialdehyde (MDA), 8-hydroxy-2’-deoxy-guanosine, NADPH oxidase, iNOS, NF-κB, STAT1, p53, caspase-3, phosphorylated mTOR, TNF-α and IL-1β -It decreased GSH, p-AMPK, p-PI3K, p-Akt less than cisplatin -Reduced: histopathological damages	[87]
N-benzylN,O-succinyl chitosan (BSCT)	356.6	−16.7	RPTEC/TERT1 cells	-Sustained and pH-dependent release	-Decreased: necrotic or late apoptotic cells	[88]
CDDP complexed with γ-polyglutamic acid and chitosan (γ-PGA/CDDP-CS)	196	−59	AB23G2-bearing mice (45 mg/kg) (i.p.)	-pH-dependent release	-Reduced: histopathological damages	[89]
Poly (lactic-co-glycolic acid) nanoparticles	284.8	−15.8	Balb/c mice (1.5 mg/kg of cisplatin in nanoparticles) (Tail-vein injection)	-A biphasic release profile	-Decreased: the cisplatin level, and CDDP-induced creatinine and urea nitrogen in kidneys	[96]
PLGA-encapsulated nano-Boldine (NBol)	115.5	−17.4	Swiss albino mice (Co-treatment of cisplatin with 10 mg/kg of NBol) (orally)	-As a nano-carrier for a natural anti-oxidant	-Recovery of SOD activity, creatinine and urea levels towards their normal ranges -Decreased: the rate of GSH depletion and the level of LPO -Normal glomerule -Negligible tubular damage	[97]
N, N’-diphenyl-1, 4-phenylenediamine loaded PLGA nanoparticles (Nano-DPPD)	–	–	Sprague–Dawley rats (0.5 g/kg) (i.p.)	-Anti-fibrotic activity	-Inhibited: the interstitial fibrosis via decreasing CDDP-induced collagen contents in kidneys -Prevented: CDDP-induced macrophages infiltration -Decreased: tubular injury score, BUN, magnesium, creatinine, MCP-1 and hydroxyproline contents -Increased: creatinine clearance	[98]
Thymoquinone nanoparticles (NP THY)	210.9	+ 32.8	Ehrlich solid carcinoma (ESC) mice model (3 mg/kg) (orally)	-Anti-oxidant and anti-inflammatory activities	-Decreased: TNF-α, IL-1β, NF-κB, urea, uric acid, creatinine, MDA and cystatin C -Increased: GSH, SOD and CAT -Preserved: parenchyma structure	[99]
Micelles of poly(ethylene glycol)-b-poly(methacrylic acid) (cisplatin/cl-micelles)	100–200	−20 to −30	C57Bl/6 mice (4 mg-cisplatin/kg b.w) (Tail-vein injection)	-Reducing renal exposure	-Recovery of creatinine and urea towards their normal ranges -No histopathological changes -No long-term effects	[101]
Polymeric micelles (CDDP-PMs) (with drug/copolymer ratios of 1:3)	18.39	−4.77	Balb/c nude mice (4 mg-cisplatin/kg) (Tail-vein injection)	-Tumor-targeting accumulation of cisplatin -A sustained, pH-dependent release	-Enhancing the dose of drug within systemic tolerability	[102]
Micellar Pluronic F127-encapsulated quercetin	–	–	Wistar rats (Co-treatment of cisplatin and 100 mg/kg of P-quercetin containing 50 mg/kg quercetin)) (i.p.)	-As a nano-carrier for a natural anti-oxidant	-Decreased: CDDP-induced urea, creatinine, and tubular damage -Increased: creatinine clearance	[105]
Chitosan derivatives, O-succinyl chitosan complexed with cisplatin	317.67	−19.23	RPTEC/TERT1 cells	–	-Decreased: cytotoxic effects of cisplatin on renal proximal tubular cells	[107]
Silymarin-loaded benzyl-functionalized succinyl chitosan (BSC) (SM-loaded PMs)	326	−23.8	RPTEC/TERT1 cells	-pH-dependent release -As a nano-carrier for silymarin as a renoprotective agent	-low cytotoxicity on renal proximal tubular cells -Increased: permeability of silymarin across the intestinal membrane	[108]
Human umbilical cord derived mesenchymal stem cells-exosomes	103	−24.15	Pre-incubated rat renal tubular epithelial cells (NRK) with exosomes	-Inhibiting apoptosis	-Increased: CDDP-inhibited viability, proliferation and G1-phase cells -Decreased: cleaved caspase-9 and -3, Bim, Bad, and Bax,	[112]
Carbon monoxide (CO)-loaded hemoglobin-vesicle (CO-HbV)	–	–	ICR mice (1000 mg Hb/kg) (Tail-vein injection)	-A renoprotectant	Suppressing caspase- 3-mediated apoptosis	[113]
Gelatin microspheres incorporating cisplatin (GM-CDDP)	–	–	Balb/c mice (GM incorporating 40 mg CDDP) (i.p.)	-Allowed high-dose chemotherapy	-Decreased: the durability of the drug in the kidney	[114]
Nanoparticles functionalized with folate (CP-FA-BSA-NPs)	134.53	−37.66	Balb/c mice (5 mg/kg of CDDP in CP-FA-BSA-NPs) (Tail-vein injection)	-Targeted cisplatin delivery	-Decreased: CDDP-induced urea, creatinine, and histopathological damage	[128]
PEG grafted-olyphosphazene–cisplatin conjugate (Polycisplatin)	18.6		ICR mice (5, 10, 15 and 20 mg platinum/kg) (i.v.)	-Passive targeting by EPR effect	-Decreased: CDDP-increased BUN, creatinine, and kidney weight/body	[116]
Poly(L-glutamic acid)-g-methoxy poly(ethylene glycol 5 K) nanoparticles	Changed based on pH	–	Kunming mice (5 mg/kg of CDDP) (i.v.)	–	-Decreased: platinum concentration in kidney	[117]
LHRH-modified dextran nanoparticles (Dex-SA-CDDP-LHRH)	–	–	Kunming mice (5 or 10 mg/kg of CDDP) (i.v.)	-Targeted cisplatin delivery	-Decreased: renal cisplatin accumulation	[118]
Gallic acid-loaded Eudragit-RS 100 Nanoparticles (nano-gallic acid)	180	Positive charge	Wistar rats (10 mg/kg) (orally)	-Anti-oxidant and anti-inflammatory activities	-Decreased: MDA and ROS production in mitochondria, mitochondrial membrane damage, TNF-α, IL-6, and histopathological damage -Increased: GSH catalase, Superoxide dismutase, and Glutathione peroxidase in mitochondria	[119]
Urolithin A nanoparticles	214	+ 25.1	C57BL/6 J mice (50 mg/kg) (orally)	-Reducing oxidative stress and apoptosis	-Attenuated: histopathological damages presented in CDDP-induced AKI -Decreased: mortality by 63%, oxidative stress, nuclear factor erythroid-2-related factor 2 (Nrf2) gene, and P53-inducible gene -Normalized: Poly(ADP-ribose) polymerase-1, miR-192-5p, miR-140-5p, intracellular NAD^+^, mitochondrial oxidative phosphorylation	[120]
A magnetic CDDP-encapsulated nanocapsule (CDDP-PAA-NC)	186	–	Balb/c nude mice (10 mg/kg) (Tail-vein injection)	-A magnetic targeting	Decreased: BUN, creatinine, and histopathological damages	[121]
Hyaluronan–cisplatin conjugate nanoparticles (HCNPs) entrapped in Eudragit S100-coated pectinate/alginate microbeads (PAMs) (HCNP-PAMs)	The diameters: 658 ± 73 µm	−20.16 for HCNPs	Wistar rats (3.5 mg/kg of cisplatin per Week) (orally)	-pH-dependent release	Decreased: creatinine level at day 29	[122]
Glutathione-scavenging poly(disulfide amide) nanoparticles (CP5)	76.2	Negative charge due to the presence of DSPE-PEG 3000 layer	A2780cis tumor bearing athymic nude mice	-Reversing CDDP resistance via GSH-scavenging process	Decreased: BUN	[123]
Hyaluronic acid cisplatin/polystyrene-polymetformin (HA-CDDP/PMet) dual prodrug co-assembled nanoparticles	166.5	−17.4	C57BL/6 mice (5 mg/kg of CDDP) (i.v.)	-Intracellular co-delivery with excellent cleavage	-Decreased: renal CDDP accumulation, BUN, creatinine, and histopathological damages	[124]
EGFR-targeted albumin-cisplatin nanoparticles	40	–	Nude mice (3.0 mg/kg equivalent Pt Dose) (Tail-vein injection)	-Targeted cisplatin delivery -A sustained, pH-dependent release	Decreased: CDDP-induced cystic dilatation of renal tubes and tubular atrophy	[125]
Pt (IV) prodrug-loaded ligand-induced self-assembled nanoparticles (GA-ALG@Pt NPs)	141.9	-36.7	Balb/c nude mice bearing dual-xenograft and Healthy Kunming mice (5 mg/kg) (Tail-vein injection) or (25 mg/kg) (i.v.)	-Redox-sensitive -Targeted cisplatin delivery	Decreased: BUN, creatinine, and tubular damage	[126]
Silk fibroin peptide/baicalein nanofibers (SFP/BA NFs):	–	–	In vitro: HK-2 cells In vivo: mice (50/100 mg/kg) (i.g.)	Improved anti-oxidant responses	Decreased: DNA damage, cGAS-STING pathway activation, creatinine, and BUN Increased: SOD	[127]
CaCO3 nanoparticle (CDDP/OA-LCC NPs)	217	−23.7	Balb/c nude mice (i.v.)	-A sustained, pH-dependent release -Anti-inflammatory activities	Decreased: NF-κB activation	[115]
Chitosan/siRNA nanoparticles	–	–	–	Passively target kidneys by gene therapy to protect them against apoptosis	Decreased: OCT1&2, p53, PKCδ and γGT proteins, and creatinine, and BUN	[92]

**Table 2:** changes in the efficiency and antitumor activity of cisplatin in the renoprotective nano-drugs

Nanoparticles	Cellular uptake	Biodistribution	Releasing efficiency	Encapsulation efficiency (EE)	Biosafety	Antitumor activity	Refs.
Tea polyphenol-functionalized SeNPs (Se@TE NPs)	Clathrin/dynamin/raft-mediated Endocytosis	–	–	–	Renoprotective	-In-vitro: stronger cell inhibition	[58]
Green silver nanoparticles (CP-AgNPs)	Increased	–	A sustainable drug release	–	- Renoprotective -Hemocompatible	-In-vitro: stronger cell inhibition	[54]
Platinum complexes of curcumin (Pt-CUR@ mPEG-SS-PBAE-PLGA)	Increased	In tumor-bearing nude mice: -Free Pt-CUR: mainly in the liver and the kidney -Nanoparticles: mainly in tumor	-Redox-and-pH-sensitive release -At pH 5.5 and reductive environment: 60% of Pt-CUR at 4 h, and 90% < at 72 h -In the presence of GSH (10 mmol/L): Pt-CUR complex could be dissociated within 4 h -Prolonged blood circulation	90.3%	-No body weight loss -Renoprotective	-In-vitro: stronger cell inhibition, apoptosis, and inhibitory effect on migration, invasion and wound healing -In-vivo: stronger inhibitory effect on tumor growth and metastasis	[74]
Liposome conjugated with EGFR antibodies (EGFR:lP-CDDP)	Receptor-mediated endocytosis	Accumulated in EGFR-expressing tumors	–	22.5%	-No body weight loss -Renoprotective -Higher survival rate	-In-vitro: stronger cell inhibition -Enhance radiosensitivity -In-vivo: stronger chemoradiotherapeutic efficacy	[78]
RNAi-Chemotherapy Layer by Layer Nanoparticle	- CD44-mediated endocytosis	–	–	18%	-Higher survival rate -Renoprotective	-In-vivo: Anti-drug resistance, and stronger inhibitory effect on tumor growth -In-vitro: stronger cell inhibition	[80]
Cisplatin-sodium alginate conjugate liposomes modified with EGF CS-EGF-Lip)	-Receptor-mediated endocytosis	EGFR-expressing tumors -Penetrated tumor spheroids	Sustained release at pH 7.4 and 5.5; with no abrupt initial release during 72	> 14%	-Less change in body weight compared to free CDDP -less nephrotoxicity	-Stronger internalization in tumor -In-vivo: stronger tumor growth inhibition -A 40.74% reduction in cancer cell migration compared to CDDP	[79]
Lipid-coated cisplatin nanoparticles	Mostly in cytoplasm and a few in lysosomes	–	A pH-responsive	80%	-No increase in serum platinum -No pulmonary toxicity, nephrotoxicity, and hepatotoxicity	-In-vivo: decreased tumor volume and weight, higher Pt uptake -In-vitro: stronger cell inhibition and apoptosis, arrested the cell cycle at G1	[85]
Curcumin-Loaded Liposomes synthesized by microfluidic technology (Lipo-Cur)	–	–	–	17 wt %	-Higher survival rate -Less nephrotoxicity	-The combination treatment of Lipo-Cur and CDDP showed a significant reduction in tumor growth -Lipo-Cur alone was as effective as CDDP	[83]
Lipocisplatin	Energy-dependent endocytosis	-Prolonged blood circulation	-Asustained pH-dependent release -At pH 7.4: 35% of CDDP is released -At pH 5.0: 75% of CDDP is released	–	-No body weight loss -Less nephrotoxicity	-Deliver more CDDP to the tumor -In-vitro: stronger cell inhibition -In-vivo: a single dose (6.5 mg Pt/kg) reduced tumor growth -In-vivo: multiple low-dose (3.5 mg Pt/kg): no tumor growth	[82]
Pt (IV) prodrug-loaded ligand-induced self-assembled nanoparticles (GA-ALG@Pt NPs)		-Pt content in liver was 3 times more than that of cisplatin -Lower content in kidney -In spleen and lung -Prolonged blood circulation	-In the presence of GSH (10 mM): they released 40% of drug within 4 h -After that: a continuous release until 120 h	Every 100 sugar units had 23.65 Pt(IV) and 9.65 GA	-No increase in serum platinum -Lower body weight loss -No nephrotoxicity and hepatotoxicity -Higher survival rate	-In-vitro: stronger cell inhibition -Pt in liver tumor was 2.5 times higher than that of lung tumor	[126]
CDDP complexed with γ-polyglutamic acid and chitosan (γ-PGA/CDDP-CS)	–	–	A pH-dependent release within 12 days (in vitro); -At pH 7.4: 9.1% of CDDP -At pH 5.5: 49.9% of CDDP	–	-Mice were survived after 13 days -Mice in CDDP group died -no nephrotoxicity	-In-vitro cytotoxicity: similar to that of free CDDP -In-vivo tumor growth inhibition: at the dose of 15 mg/kg, it was similar to that of free CDDP	[89]
N-benzylN,O-succinyl chitosan (BSCT)	Increased	–	-pH-dependent release -A rapid release within 4 days -A sustainable release on days 5 to 7	-EE: 48.69% -loading capacity: 0.74	–	-In-vitro: stronger cell inhibition, and increased early apoptosis -the release of CDDP at 8–24 h induced killing of cancer cells	[88]
Carbon monoxide (CO)-loaded hemoglobin-vesicle (CO-HbV)	–	–	–	–	-Renoprotective -Less body weight loss -Higher survival rate	-In-vitro: weak anti-tumor activity -In-vivo: tumor growth inhibition	[113]
Chitosan derivatives, N-octyl-N,O-succinyl chitosan (OSCS) complexed with cisplatin	–	–	–	-EE: 64.50% -Loading capacity: 0.28%	-Renoprotective	Good anti-cancer activity, but less than free CDDP	[107]
Micelles of poly(ethylene glycol)-b-poly(methacrylic acid) (cisplatin/cl-micelles)	Macrophage capture	-Accumulation in kidney, liver and spleen -Prolonged blood circulation	-Lower release rate in blood -Higher rate in tumors	-Loading capacity: 30% w/w	-No hemolytic activity -Higher survival rate -No body weight loss -Renoprotective	-Higher tumor platinum levels -In-vivo: stronger tumor growth inhibition	[101]
Polymeric micelles (CDDP-PMs) (with drug/copolymer ratios of 1:3)	–	-In liver and spleen -Prolonged blood circulation	-pH-dependent release over 10 days	-EE: 92.43% -Drug loading: 21.24	-Renoprotective -No body weight loss	-In-vitro: higher early apoptosis (concentration dependent) -Higher tumor platinum levels than CDDP -In-vivo: lower tumor growth inhibition than CDDP -Improve the tolerance to higher dose of CDDP	[102]
Silymarin (SM)-loaded polymeric micelles	–	–	-pH-sensitive release -At pH 7.4: Rapid release in 4 h	47–70%	Renoprotective	-In-vitro: increased killing efficacy	[108]
Gelatin microspheres incorporating cisplatin (GM-CDDP)	–	-Fewer Pt level in serum and ascites within 4 h -CDDP was detectable within 168 h, while it reduced rapidly in free CDDP	–	–	-Less hematotoxicity and nephrotoxicity -Higher survival rate -No weight loss	-In-vivo: tumor weight was similar to that of free CDDP	[114]
Poly (lactic-co-glycolic acid) (PLGA) nanoparticles	–	-Higher platinum level in liver -Lower platinum level in kidney, spleen and lungs	-On the 1^st^ day: an initial burst release -After that: a controlled release	-EE: 3.0% (w/w) -Drug loading: 1.01 μg/mg	Renoprotective	-In-vitro: almost similar anti-proliferative activity	[96]
Thymoquinone nanoparticles	–	–	–	-EE: 96% -Drug loading: 10.2%	-Renoprotective -Survival rate and weight loss were similar to CDDP	-In-vivo: reduced tumor weight similar to CDDP	[99]
Aptamer functionalized cisplatin-albumin nanoparticles	-Receptor-mediated endocytosis -EGFR positive cells	-In EGFR positive tumors -Lower Pt accumulation in heart, liver, spleen, and kidney than CDDP -Less clearance by RES	At pH 5.5: a sustained release over 72 h	–	-No weight loss -Renoprotective -Normal spleen, heart and liver	-In-vitro: stronger cell inhibition, apoptosis and necrosis -In-vivo: stronger tumor growth inhibition, and higher apoptosis rate -In-vivo: reduced tumor weight	[125]
Poly(L-glutamic acid)-g-methoxy poly(ethylene glycol 5 K) nanoparticles	–	–	-Slower release of CDDP	–	-Hemocompatible -Renoprotective -Less weight loss -Higher survival rate	-In-vitro: lower cytotoxicity -In-vivo: tumor growth inhibition, but less potent than CDDP	[117]
PEG grafted-olyphosphazene–cisplatin conjugate (Polycisplatin)	Targeted tumor by EPR effect	-24 h after injection: mostly in tumor -A time-dependent reduction in plasma	-At pH 7 and pH 5: a 50% release within 1 day and a 60% release within 2 weeks	–	-No body weight loss -Renoprotective -Higher survival rate	-In-vivo: at the dose of 1.95 mg Pt/kg tumor growth inhibition similar to CDDP, but stronger effect at 3.9 mg Pt/kg	[116]
Hyaluronic acid cisplatin/polystyrene-polymetformin (HA-CDDP/PMet) dual prodrug co-assembled nanoparticles	CD44-receptor mediated endocytosis and EPR effect	-Mostly in tumor -Less than free drugs in kidney	pH-dependent release: -At pH 7.4: sustained release of CDDP and no MET release during 48 h -At pH 5.5: fast CDDP release	For CDDP and metformin: 3.7% and 15.2%, respectively	-Higher survival rate -Renoprotective -No body weight loss	-In-vitro: stronger cell inhibition and apoptosis -In-vivo: excellent CDDP and metformin cleavage -Deliver more CDDP to the tumor -In-vivo: stronger tumor growth inhibition	[124]
Hyaluronan–cisplatin conjugate nanoparticles (HCNPs) entrapped in Eudragit S100-coated pectinate/alginate microbeads (PAMs) (HCNP-PAMs)	–	–	pH-dependent release: -At pH 1.2: 25.1% of HCNPs during 24 h -At pH 4.5: 39.7% -At pH 7.4: 75.6%	-CDDP content: 16.1% - Loading efficiency: 80.6%	-Renoprotective -No body weight loss	-In-vitro: stronger cell inhibition	[122]
A magnetic CDDP-encapsulated nanocapsule (CDDP-PAA-NC)	Higher internalization by magnetic targeting	-Mostly in tumor -Prolonged blood circulation and the removal of Pt by kidney -Lower Pt level in liver and spleen	-42.2% in 24 h	-PAA:CDDP ratio = 2:1 -EE: 83.4% -Loading capacity: 6.09%	-No toxicity on spleen, liver and kidney	-Deliver more CDDP to the tumor -In-vivo: stronger tumor growth inhibition	[121]
Urolithin A nanoparticles	–	Enhanced oral bioavailability of UA	–	~ 35%	-Higher survival rate -Less weight loss -Renoprotective	–	[120]
Nanoparticles functionalized with folate (CP-FA-BSA-NPs)	Receptor mediated-endocytosis	–	-At pH 7.4 and 37 °C: A rapid initial release, then a sustainable release	-EE: 86.86% -Drug loading 43.43 μg/mg nanoparticle	-Renoprotective	-Deliver more CDDP to the tumor	[128]
LHRH-modified dextran nanoparticles (Dex-SA-CDDP-LHRH)	Receptor mediated-endocytosis	-In normal mice: compared to free CDDP, NPs induced greater Pt accumulation in liver, spleen and lung, and lower in kidney -In tumor-bearing mice: NPs mostly accumulated in tumors, induced greater Pt accumulation in liver, spleen and lung -Prolonged blood circulation	–	–	-Renoprotective -Higher survival rate -No body weight loss	-Deliver more CDDP to the tumor -In-vitro: stronger cell inhibition -Improved maximum tolerated dose of CDDP -Higher antimetastasis effects -In-vivo: stronger tumor growth inhibition	[118]
Cysteine prodrug 5 NPs	Higher uptake In cisplatin-resistant cells with upregulation of GSH	–	At pH 7.4: -In a 10 mM DTT solution: 80% over 72 h -In a 1 mM DTT solution: 30% -without DTT: < 10%	15.50 Pt%	-Renoprotective -No body weight loss -No toxicity on liver and spleen -Minimal hemolysis	-In-vitro: stronger cell inhibition and apoptosis in cisplatin-resistant cells -In-vivo: stronger tumor growth inhibition -reduced cisplatin-resistance in cells with upregulation of GSH	[123]
CaCO3 nanoparticle (CDDP/OA-LCC NPs)	EPR effect	-Prolonged blood circulation -Mostly in tumor -lower accumulation in kidney	pH-dependent release: -At pH = 5.5: 70% after 72 h -At pH 7.4: 28%	Drug loading: 76%	-Renoprotective -No body weight loss	In-vitro: stronger cell inhibition -In-vivo: stronger tumor growth inhibition and apoptosis -Deliver more CDDP to the tumor	[115]

To design new CDDP-based nano-carrier with less nephrotoxic effect, this study aimed to review some of the nanotechnology-based methods that have been able to alleviate the CDDP-induced nephrotoxicity. For this purpose, some of the main nephrotoxic mechanisms of cisplatin are firstly introduced and then the applications of both metallic and polymeric nanoparticles in reduction of the CDDP-associated nephrotoxicity were reviewed between 2011 and 2022.

### The main mechanisms involved in CDDP-induced nephrotoxicity

Histologically, cisplatin can cause some dose–response changes in the collecting ducts, distal tubules, and most importantly, in the S3 segment of the proximal tubules of renal tissue (see details in Table 3).

**Table 3:** The in-vivo effects of cisplatin and nanoparticles on renal histopathology

Nanoparticles		Cisplatin	
Name	Effects		
Cerium oxide nanoparticles (CONPs	Mild tubular cloudy swelling and necrosis	• Tubular apoptosis, necrosis, intratubular eosinophilic material, and atrophy	[52]
Nanoceria	• Inhibition of CDDP-induced kidney weight reduction after acute (14 days) and chronic (28 days) exposure, • Normal tubules	• Tubular dilation • Reduced kidney weight	[53]
Green silver nanoparticles (CP-AgNPs)	• Tubules: mild cloudy swelling, cast formation, regenerated tubular epithelium, vacuolation • Glomeruli: few shrunken, wide Bowman’s spaces, periglomerular leukocytic infiltration	• glomerular lesions: congestion, collapse, atrophy, and necrosis • tubular lesions: necrosis of the lining epithelium, extensive vacuolation, atrophy, cystic dilatation, cast formation • Interstitium: vascular congestion, leukocytic infiltration	[54]
N-(2-hydroxyphenyl) acetamide-conjugated gold nanoparticles (NA2-AuNPs)	• A great ameliorating effects on kidney damage scores at the dose of 25 mg/kg • Normal renal cortex, corpuscles and tubules • Protection of brush borders of proximal convoluted tubules	• Excessive epithelial vacuolization, • Glomerular atrophy • Damaged brush borders	[55]
selenium nanoparticles (SeNPs)	• Fish oil + SeNPs + CDDP + γ-radiation group: surface blebs, swelling of tubular occasional cells, intratubular eosinophilic material	• CDDP group: Glomerular lesions: atrophy, fibrosis, thick basement membrane, wide Bowman’s space. Tubular lesions: necrosis and edema in convoluted tubules, hemorrhage • CDDP + γ-radiation group: More severe hemorrhage, glomerular atrophy, dilated tubules, leucocytic infiltration	[57]
Tea polyphenol-functionalized SeNPs (Se@TE NPs)	Normal	• Swelling of tubular cells and glomeruli • Narrowing interstitial space	[58]
Se@SiO2 nanocomposites	Normal	Tubular epithelial injury, inflammatory infiltration, cast formation, tubular dilatation	[60]
A. officinarum-silver nanoparticles (AG-AO)	Normal	Tubular atrophy, necrosis, cortex and medullary damage, Interstitial edema and inflammation	[62]
Curcumin nanoparticles	• Normal • Proximal tubular area = 1500 μm^2^	• Tubular necrosis (mild to moderate), proteinaceous cast formation, • Proximal tubular area = 893 μm^2^	[73]
Curcumin nanoparticles	Normal	• Shrinkage of the glomerulus, wide Bowman’s space • Tubular atrophy, necrosis, cast formation	[72]
Cisplatin-incorporated liposome conjugated with EGFR antibodies (EGFR:lP-cDDP)	Normal	Acute cortical tubular degeneration and regeneration	[78]
RNAi-Chemotherapy Layer by Layer Nanoparticle	Normal	Swollen tubules	[80]
Curcumin-Loaded Liposomes synthesized by microfluidic technology	Normal	Acute tubular necrosis, multiple necrotic cells, debris in lumen,	[83]
Nanoparticulated honokiol	Reduced CDDP-induced histopathological changes	Inflammatory infiltration, interstitial fibrosis, tubular lesions, smaller kidneys with pale appearance,	[84]
Lipid-coated cisplatin nanoparticles	Normal	• Collapsed glomeruli; mesangial cells fused with tubules, dilated arteries with red blood cells • Systemic injection group: collapsed Bowman’s capsule, and disappeared Bowman’s space • Local injection group: lymphocyte infiltration	[85]
Cisplatin NanoComposite (CHIT/Cis/MgO NPs)	Normal structure with congested intertubular blood vessels and albuminous casts	• Massive intertubular hemorrhage, tubular degenerative changes, extended renal tubules, leukocyte infiltration, necrosis • Glomerular atrophy, wide Bowman’s space	[87]
CDDP complexed with γ-polyglutamic acid and chitosan (γ-PGA/CDDP-CS)	Normal tubules, but edematous mesenchymal tissues	Damage to proximal tubules	[89]
poly (lactic-co-glycolic acid) nanoparticles	Normal	Pathological atrophy	[96]
PLGA-encapsulated nano-Boldine (NBol)	• Normal glomerular structure • Minimal tubular damage	Glomerular atrophy, tubular damage, dilatation of urinary space	[97]
N, N’- diphenyl-1, 4-phenylenediamine loaded PLGA nanoparticles (Nano-DPPD)	Decreased fibrosis score and tubular injury score	Increased the scores	[98]
Thymoquinone nanoparticles (NP THY)	• Almost normal parenchyma structure with negligible deformity of few corpuscles • Normal tubules with some luminal dilation in cortex • Infiltrated neoplastic cells: degenerated	• Disorganization of parenchyma • Atrophy and deformity of corpuscle/glomeruli • Degenerative changes in tubule lining • Epithelium: e.g., deformed small nuclei, and swollen cells • Infiltrated neoplastic cells: degenerated	[99]
Cisplatin/cl-micelles	• After 13 days: normal • After 28 days: reduced CDDP-induced histopathological changes by regenerating tubules in cortical subcapsular region, but some change, e.g., basophilic tubules and interstitial lymphocytic infiltrate, were irreversible	• After 13 days: all animals: tubular vacuolization, basophilia; some animals: necrosis in tubules and collecting duct, and reduced cellularity in papilla	[101]
P-quercetin	• Reduced cortical injury • No impact on medullary injury	• Massive tubular necrosis in outer medulla, and slight damage in cortical region • Tubular dilation, increased hyaline material	[105]
Carbon monoxide (CO)-loaded hemoglobin-vesicle (CO-HbV)	Normal	• Tubular injuries: swelling and detachment	[113]
Nanoparticles functionalized with folate (CP-FA-BSA-NPs)	Normal	• Reduced kidney weight • After 1 week: necrosis	[128]
LHRH-modified dextran nanoparticles (Dex-SA-CDDP-LHRH)	Reduced CDDP-induced histopathological changes	• Severe necrosis in proximal tubules • Loss of tubular brush border • Thick glomerular basement membrane and mesangium	[118]
nano-gallic acid	• At concentration of 10 mg/kg: normal • At concentration of 50 and 100 mg/kg: proximal tubule injury, infiltration of inflammatory cell and RBCs	More severe than NPs induced tubular injury and inflammatory cells infiltration	[119]
Urolithin A nanoparticles	Reduced CDDP-induced histopathological changes	Interstitial expansion, tubular necrosis, atrophy, dilation, apoptotic bodies, casts, thicker glomerular basement membranes	[120]
A magnetic CDDP-encapsulated nanocapsule (CDDP-PAA-NC)	Normal	Swollen proximal tubules, vacuolar degeneration/dilation, basophilic tubules in cortex	[121]
EGFR-albumin-cisplatin nanoparticles	Normal	Tubular atrophy and cystic dilatation	[125]
Pt (IV) prodrug-loaded ligand-induced self-assembled nanoparticles (GA-ALG@Pt NPs)	Normal	• In corticomedullary region: acute proximal tubular injury • Tubular dilation, hydropic degeneration, and protein casts	[126]
Nanocomposites of CDDP-chitosan	• Normal glomeruli, slightly wider capsular space • Normal tubular epithelial lining	• Glomerular and tubular damages	[91]
CaCO3 nanoparticle (CDDP/OA-LCC NPs)	• Almost normal	• Loss of brush border • Loss of epithelium • Tubular necrosis	[115]

Among all three isoforms of the organic cation transporters (OCTs), OCT2 is probably involved in the renal absorption of cisplatin [26, 27]. Moreover, both copper transporter receptor 1 (CTR1), which is expressed in the proximal tubules, and the multidrug extrusion transporter-1 (MATE1), as a cisplatin efflux transporter, are also involved in cisplatin uptake of renal cells [9, 28, 29]. Upon entry, CDDP is hydrolyzed and GSH is conjugated to the cationic derivatives of cisplatin through the glutathione S-transferases (GST) [30, 31]. Subsequently, γ-Glutamyl Transferase (GGT) converts these conjugates to cysteinyl-glycine-conjugates, which are more cleaved to cysteine-conjugates by alanine aminopeptidase [32, 33]. In the proximal tubular epithelial cells (PTECs), these conjugates produce nephrotoxins [27, 28]. Hence, the use of GGT inhibitors, such as aminooxyacetic acid, can be a protective mechanism.

On the other hand, by forming DNA adducts in both mitochondrial and nuclear DNA, cisplatin can induce either apoptosis at low doses (8 μM) or necrosis at higher doses (800 μM) [34]. The cells with more mitochondria, such as PTECs, are more sensitive to cisplatin, as the negatively charged mitochondria membrane leads to a greater tendency of mitochondrial DNA to interact with these derivatives [35–37].

**Fig. 2:**
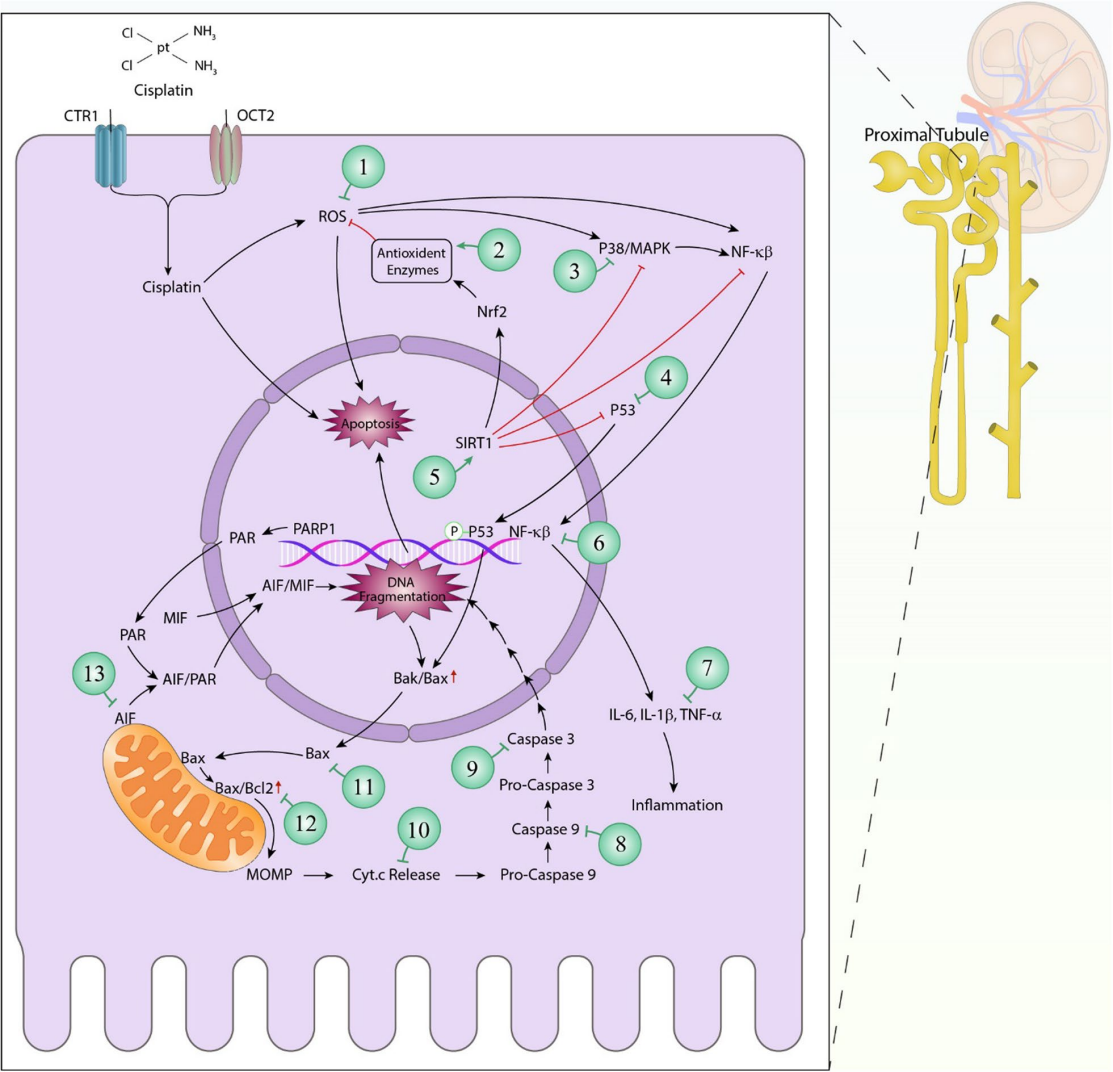
Cisplatin induced-nephrotoxicity signaling pathways upon entering a renal Proximal tubular epithelial cell versus the ameliorative effects of metallic nanoparticles. Each green circle is a representative of a nanoparticles' group that may have a positive or Ameliorative effect on the selected signaling element. (1) Cerium oxide nanoparticles (CONPs), Tea polyphenol-functionalized Selenium NPs (Se@TE), A. officinarum-silver NPs (AG-AO; (2) CONPs, N-(2-hydroxyphenyl) acetamide-conjugated gold NPs (NA2-AuNPs), AG-AO; (3) Se@TE; (4) Se@TE, 6-hydroxy-2,5,7,8-tetramethylchroman-2-carboxylic acid (trolox) surface-functionalized selenium nanoparticles (Se@Trolox), AG-AO; (5) Se@SiO2 nanocomposites; (6) NA2-AuNPs, AG-AO; (7) CONPs, NA2-AuNPs, SeNPs, Se@SiO2 nanocomposites, AG-AO; (8) AG-AO; (9) cisplatin-loaded green silver nanoparticles (CP-AgNPs), SeNPs, Se@TE, AG-AO; (10) CP-AgNPs; (11) CP-AgNPs, Se@TE, AG-AO; (12) Se@TE, AG-AO; (13) CP-AgNPs. Organic cation transporters 2 (OCT2); Copper transporter receptor 1 (CTR1); Reactive oxygen species (ROS); Nuclear factor erythroid related factor 2 (Nrf-2); MAPK (Mitogen-activated protein kinase); Nuclear factor kappa β (NF-κβ); Sirtuin 1 (Sirt1); poly ADP-ribose polymerase-1 (PARP1); Macrophage migration inhibitory factor (MIF); Activates apoptosis-inducing factor (AIF); Bcl-2 associated X-protein (BAX); B-cell lymphoma 2 (Bcl-2); Mitochondrial outer membrane permeabilization (MOMP); Cytochrome complex (cyt.c); Interleukin (IL); Tumor necrosis factor α (TNF-α)

**Fig. 3:**
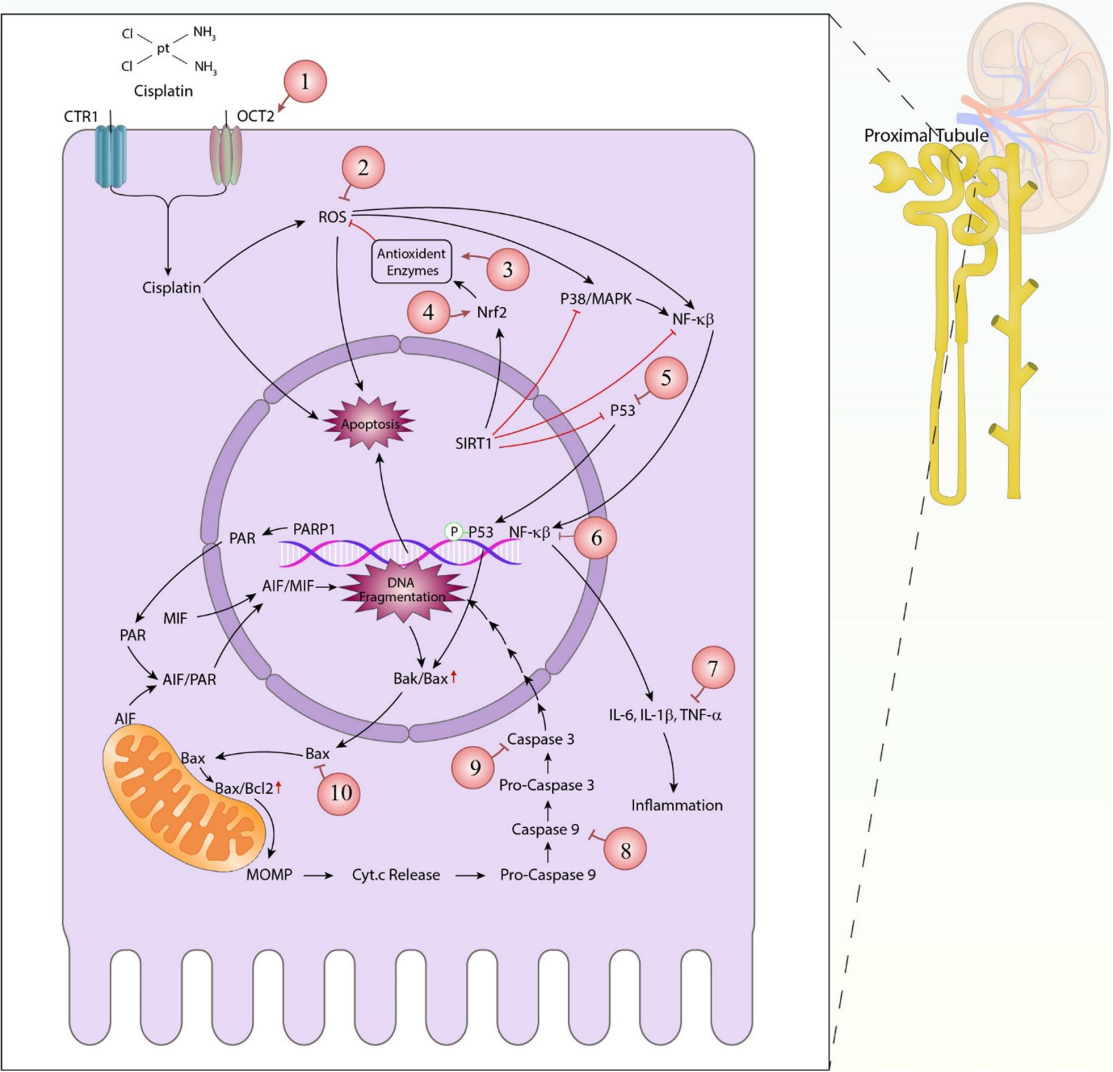
Cisplatin induced-nephrotoxicity signaling pathways upon entering a renal Proximal tubular epithelial cell versus the ameliorative effects of polymeric nanoparticles. Each green circle is a representative of a nanoparticles' group that may have a positive or ameliorative effect on the selected signaling element. (1) Nanocurcumin; (2) The platinum complexes of curcumin (Pt-CUR@ mPEG-SS-PBAE-PLGA), Gallic acid-loaded Eudragit-RS 100 NPs; (3) Nanocurcumin, Thymoquinone nanoparticles (NP-THY), Gallic acid-loaded Eudragit-RS 100 NPs; (4) Urolithin A nanoparticles; (5) Cisplatin NanoComposite (CHIT/Cis/MgO NPs), Urolithin A nanoparticles; (6) CHIT/Cis/MgO NPs, NP-THY; (7) Nanocurcumin, CHIT/Cis/MgO NPs, NP-THY, Gallic acid-loaded Eudragit-RS 100 NPs; (8) Exosomes; (9) Nanoparticulated honokiol, CHIT/Cis/MgO NPs, Exosomes, Carbon monoxide (CO)-loaded hemoglobin-vesicle (CO-HbV); (10) Exosomes. Organic cation transporters 2 (OCT2); Copper transporter receptor 1 (CTR1); Reactive oxygen species (ROS); Nuclear factor erythroid related factor 2 (Nrf-2); MAPK (Mitogen-activated protein kinase); Nuclear factor kappa β (NF-κβ); Sirtuin 1 (Sirt1); poly ADP-ribose polymerase-1 (PARP1); Macrophage migration inhibitory factor (MIF); Activates apoptosis-inducing factor (AIF); Bcl-2 associated X-protein (BAX); B-cell lymphoma 2 (Bcl-2); Mitochondrial outer membrane permeabilization (MOMP); Cytochrome complex (cyt.c); Interleukin (IL); Tumor necrosis factor α (TNF-α)

The CDDP-induced apoptosis is mediated by different signaling pathways in kidneys (as summarized in Figs. 2 and 3). Firstly, the caspase-independent and caspase-dependent pathways are triggered to induce pro-apoptotic responses. In independent pathways, poly (ADP-ribose) polymerase-1 (PARP1), which is a nuclear factor involved in DNA repair, activates apoptosis-inducing factor (AIF) on the mitochondrial membrane. As a result, this factor will be translocate to the nucleus and induce apoptosis [38]. Therefore, the inhibition of PARP1 can be proposed as a therapeutic strategy [39]. The apoptotic response may also be due to Bcl/Bax and caspases signaling. In this pathway, Bax translocates from the cytosol to the mitochondria and induces cytochrome c release. Subsequently, the final activations of caspase-9 and caspase-3 lead to apoptosis [40, 41]. Moreover, p53, as a tumor suppressor protein, may also be involved in cisplatin-induced apoptosis in the kidney. In this regard, after damaging DNA, this protein is phosphorylated, which in turn stimulates pro-apoptotic proteins. Therefore, the inhibition of p53 in renal cells may be considered as a renal protective strategy [42]. Finally, cisplatin can also bind to several death receptors such as tumor necrosis factor receptor 1 and 2 (TNFR1 and TNFR2) and Fas receptor (FasR), and activate caspases-8 and 3 [43].

By acting on cytochrome P450 in the ER membrane, cisplatin also induce oxidative stress and apoptosis [44]. To induce oxidative stress, this drug may also react with antioxidant enzymes (e.g., catalase [CAT]) and reduces their bioavailability in cells [45, 46]. In the following, reactive oxygen species (ROS) can affect the electron transport chain of mitochondria, which inhibit adenosine triphosphate (ATP) production, and induce lipid peroxidation and cell membrane permeability [43]. Moreover, cisplatin-induced hydroxyl radicals can activate the nuclear factor kappa-light-chain-enhancer of activated B cells (NF-κB) signaling pathway in renal cells by affecting the p38/mitogen-activated protein kinase (MAPK) pathways and increasing tumor necrosis factor α (TNF-α) expression [47]. This mechanism produces pro-inflammatory cytokines as well as their adhesion molecules, e.g., intercellular adhesion molecule 1 (ICAM1), in kidneys, leading to renal failure and apoptosis. The inhibition of this process may also be a renal productive method [48]. Epigenetic modifications can also affect cisplatin-induced nephrotoxicity as previously described by Loren et al. [49].

In general, the main nephrotoxic effects of cisplatin were introduced in this section and shown at Figs. 2 and 3).

### Nephroprotective approaches based on nanotechnology

In the following, the capabilities of nanotechnology in reducing cisplatin-induced nephrotoxicity and improving cisplatin efficiency for an antitumor activity are described. The results are summarized in Tables 1, 2, 3 and Fig. 1.

## Metallic nano-formulations

Some cisplatin-loaded nanoparticles can reduce CDDP-induced nephrotoxicity. One of these nanocarriers is cerium oxide nanoparticles (CONPs) that act as a free radical scavenger to regulate the antioxidant system. Depending on the oxidative state of the cells, Ce^4+^/Ce^3+^ redox partner on the surface of CONPs can act as cellular metallo-enzymes to regulate oxidative stress [50]. In mimicking the role of SOD, Ce^3+^ can be oxidized into Ce^4+^. As a result, superoxide is reduced into hydrogen peroxide. These nanoparticles can also scavenge hydroxyl radical, peroxynitrate and nitric oxide (NO) from the cell. The conversion of Ce^4+^ to Ce^3+^ can oxidize hydrogen peroxide to O_2_ [51]. Thus, these mechanisms can help restore and repair the antioxidant system after taking cisplatin.

In this regard, the combination treatment of adult male albino rats with cisplatin (for 3 weeks) and CONPs (daily for 4 weeks) reduced the kidney injury markers. Moreover, despite the fact that CDDP could reduce anti-inflammatory IL-10 cytokine and total antioxidant (TAO) in kidneys, these nanoparticles could restore anti-inflammatory and antioxidant responses to protect renal tissue [52]. Another study revealed that the injection of CONPs into Swiss albino mice could reduce the histopathological effects of cisplatin in the kidney after both acute and chronic exposures. Indeed, these nanoparticles (NPs) could reduce lipid peroxidation and pro-inflammatory cytokines, such as IL-6 and TNF-α, and increase antioxidants, such as CAT and GSH, in cisplatin-treated mice [53].

In addition to CONPs, the load of cisplatin in silver nanoparticles (AgNPs) could also be helpful. These nano-drugs not only improved the drug efficiency of cisplatin on prostate cancer cell line, but also ameliorated CDDP-induced nephrotoxicity. It was shown that CDDP-loaded AgNPs could inhibit mitochondria-mediated apoptosis in the kidney via decreasing the mRNA expression of pro-apoptotic proteins, e.g., Bax and caspase-3, and increasing the expression of anti-apoptotic proteins, e.g., Bcl-2, in treated Wistar rats. In fact, the AgNPs inhibited the release of AIF and cytochrome c from mitochondria via reducing the activation and translocation of Bax into mitochondrial membranes, which in turn prevents caspase-dependent apoptosis in kidneys. Interestingly, the renal tissue of mice treated with AgNPs was almost normal [54].

Another way to reduce cisplatin-induced nephrotoxicity is to use nanocarriers that can deliver antioxidants and anti-inflammatory substances to the kidneys by increasing their bioavailability. In one study, N-(2-hydroxyphenyl) acetamide-conjugated gold nanoparticles (NA2-AuNPs) were used to reduce the nephrotoxic effects of cisplatin in male Balb/c mice. After pretreatment of mice by intraperitoneal injection of different concentrations of NA2-AuNPs for 5 days, a single dose of cisplatin was injected. After 72 h, the urea and creatinine were reduced in NP-pretreated group in a dose-dependent manner compared to CDDP group. Furthermore, this study showed that NA2-AuNPs were able to downregulate the mRNA expression of cisplatin-induced NF-κB, inducible nitric oxide synthase (iNOS) and IL-6, and upregulate hemeoxygenase-1 (HO-1) gene expression [55]. In another study, different concentrations of Ficus carica L. leaves extract-loaded AuNPs could successfully reduce the cisplatin-induced acute kidney injury (AKI) in albino rats. This plant is rich in phenolic compounds that gives it an excellent antioxidant property. The NPs/Fig leaves extract ratio of 3/2 could significantly reduce malondialdehyde (MDA), hydroxyproline, urea, creatinine and homocystein compared to CDDP [56].

The use of selenium nanoparticles (SeNPs) in the manufacture of antioxidants has also received much attention. In this regard, Saif-Elnasr et al. investigated the effect of pretreatment of mice with both SeNPs and fish oil on CDDP-induced renal toxicity after a combination radiotherapy–chemotherapy protocol. Despite the fact that the combination of cisplatin with radiotherapy may exacerbate its cytotoxic effects, this study was able to reduce the nephrotoxic effects by inhibiting caspase-dependent apoptosis and inhibiting inflammation, and strengthening the antioxidant system [57]. Furthermore, another study used tea polyphenol-functionalized SeNPs (Se@TE NPs) to reverse the nephrotoxic effect of cisplatin on HK-2 cells and KM mice in a concentration dependent manner. Tea polyphenols (TP) is a polyphenolic plant with antioxidant-antitumor properties. While Se@TE NPs were cytotoxic for cancer cells, they had no adverse effect on normal HK-2 cells. The nanoparticles entered the renal cells via the endocytosis-mediated clathrin/dynamin/raft pathway. The study of signal transduction pathways, i.e., MAPK and phosphoinositide 3-kinase/protein kinase B (PI3K/AKT), revealed that although CDDP increased the levels of dephosphorylated AKT, phosphorylated p38 MAPK and phosphorylated c-Jun N-terminal kinase (JNK), co-incubation of it with Se@TE NPs could mitigate these impacts. These nanoparticles could dose-dependently inhibit caspase-induced apoptosis in cisplatin-treated HK-2 cells. Moreover, Se@TE NPs prevented mitochondrial dysfunction via upregulating anti-apoptotic (i.e., Bcl2 and Bcl-xL) and downregulating pro­apoptotic (i.e., Bax and Bad) genes. Also, these nanoparticles were able to inhibit p53 phosphorylation and DNA damage by inhibiting cisplatin-induced ROS in kidneys. Since selenium can be attached to Se-containing amino acids, e.g., Selenomethionine (Se-Met) and Selenocysteine (Se­Cys), it has been suggested that the Se@TE NPs can inhibit ROS production of cisplatin through activating seleno-enzymes like glutathione peroxidase (GSH-Px) [58]. Another study used 6-hydroxy-2,5,7,8-tetramethylchroman-2-carboxylic acid (trolox) as a capping agent for the synthesis of 100 nm SeNPs. Trolox surface decoration could prepare a more compact and stable globular SeNPs. These nanoparticles could inhibit apoptosis via blocking ROS-induced p53 phosphorylation and regulating AKT/MAPK pathways in renal cells [59]. In addition, Li et al. were able to use 53 nm Se@Sio2 nanospheres to reduce cisplatin-induced AKI in both HK-2 cells and C57BL/6 mice. These studies revealed that pretreatment of mice with these nanoparticles reversed cisplatin-induced tubular damage in mice by reducing the TNF­α and IL-6 overexpression by cisplatin. Moreover, these nanospheres activated Sirtuin 1 (Sirt1) at both in-vivo and in-vitro studies [60]. Previous data had already shown that an increase in Sirt1 level was associated with a reduction in the nephrotoxic effects of cisplatin [61]. The reason is that Sirt1 prevents mitochondrial mediated apoptosis by regulating Bcl/Bax ratio. Hence, the knockdown of Sirt1 could totally inhibit this anti­apoptotic effect of SeNPs [60].

In addition, the green synthesis of AgNPs of *Alpinia officinarum*, as a natural antioxidant, could reduce the nephrotoxic effects of cisplatin on AgNPs­treated Wistar rats. Like other metallic nanoparticles, these NPs also decrease cisplatin-induced ROS levels, and both pro­apoptotic and pro­inflammatory responses in kidneys. In this regard, they could also trigger the antioxidant systems (e.g., superoxide dismutase (SOD), CAT and GSH), reduce Bax, p53, caspase­3 and ­9 proteins, and increase Bcl2 protein. Also, these NPs mitigated pro-inflammatory cytokines, e.g., TNF­α and IL­1β, by reducing NF-κB and NO pathways. Interestingly, the severity of tubular atrophy, interstitial edema, necrosis and inflammation was significantly reduced in AgNPs­treated rats compared to cisplatin-treated rats [62]. Previous studies had shown that diarylheptanoids groups in *Alpinia officinarum* can attenuate NO production in mice, and its phenolic hydroxyl agents enable to regenerate antioxidant [63]; therefore, the AgNPs may improve these effects through increasing their bioavailability [62].

Overall, the metallic nanoparticles can induce the ameliorative effects on nephrotoxicity of CDDP either using their innate antioxidant/anti-inflammatory activities or carrying antioxidant/anti-inflammatory agents (see details in Table 1 and Fig. 2).

## Polymeric nano-formulations

### Curcumin nanoparticles

Curcumin is the active compound of *Curcuma longa* which is renoprotective and has antioxidant, chemotherapeutic and anti-inflammatory impacts [64, 65]. This compound can reduce the nephrotoxic effect of cisplatin by reducing the expression of OCT2 in renal cells [66]. Besides, the phenolic group in the structure of curcumin can inhibit oxidative stress via overexpressing HO-1, GST and NAD(P)Quinine oxidoreductase1 (NQO1), and acting as a ROS scavenger [67–69]. Therefore, the combination of cisplatin with curcumin may be considered as a nephroprotective approach. However, curcumin has some significant drawbacks, some of which are low water-solubility, instability at physiological pH, low absorption and poor bioavailability [70, 71]. To solve such problems, curcumin nanoparticles, with dimensions of 10–100 nm, can be useful. The larger surface area of nanoparticles, which increases the dissolution, can enhance the aqueous solubility and bioavailability of curcumin [72].

**Fig. 4:**
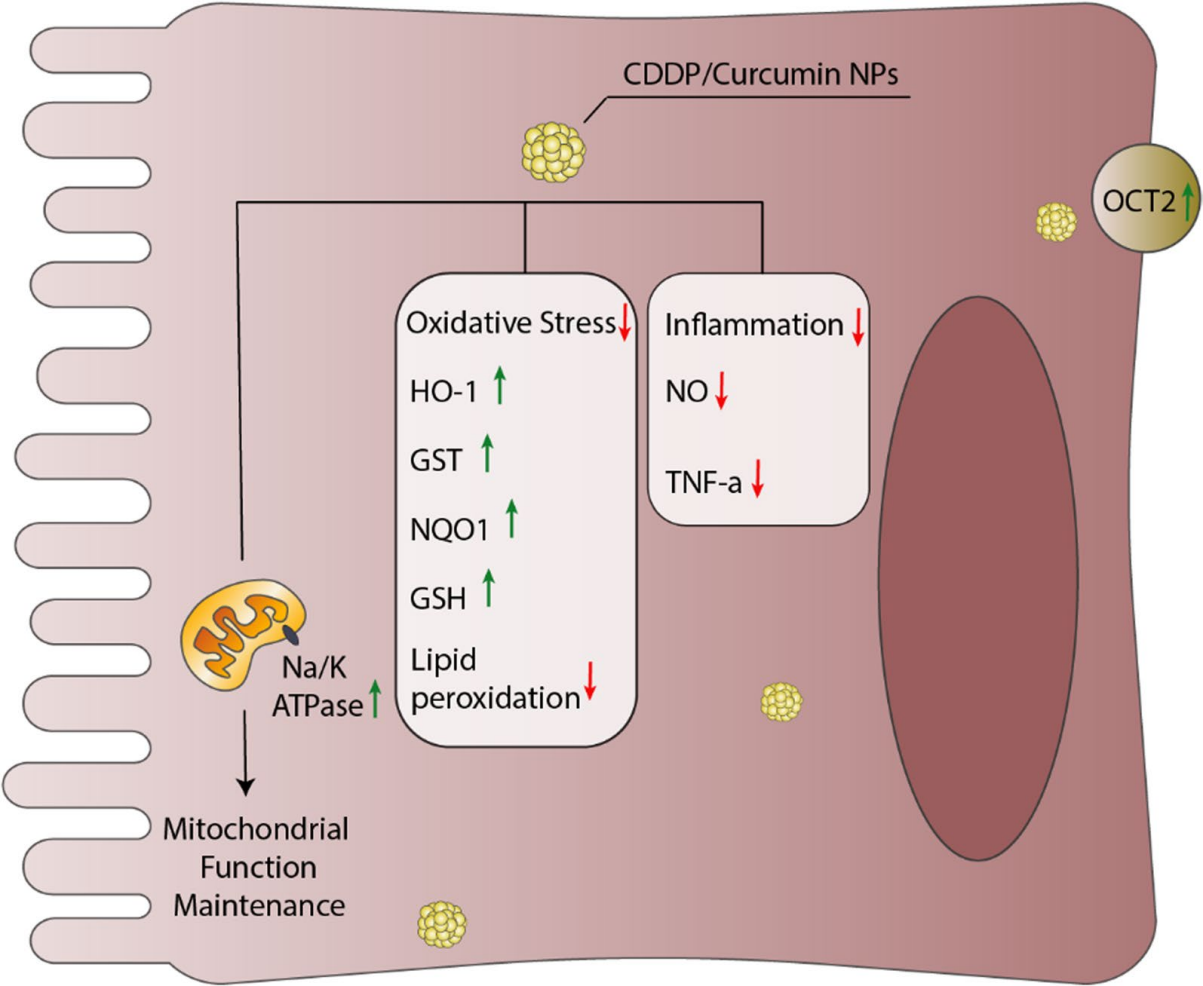
The amilorative effects of CDDP Curcumin nanoparticles in renal cells. By increasing the expression of OCT2, Curcumin NPs can mitigate the nephrotoxic effects of cisplatin. Furthermore, Curcumin reduces inflammation by decreasing concentration of NO and TNF-α. It also decreases oxidative stress By overexpressing HO-1, GST, NQO1, serving as a ROS scavengers, and GSH. It improves mitochondrial function by rising Na + /K + -ATPase activity. Cisplatin-diamminedichloroplatinum (CDDP); Organic cation transporters 2 (OCT2); Nitric oxide (NO); Tumor necrosis factor α (TNF-α); Hemeoxygenase-1 (HO-1); glutathione S-transferases (GST); NAD(P)Quinine oxidoreductase1 (NQO1); Glutathione (GSH)

The effects of curcumin nanoparticles on CDDP-induced nephrotoxicity have been illustrated in Fig. 4. Curcumin nanoparticles could reduce lipid peroxidation, NO and TNF-α productions in the kidneys of orally treated rats following a single intraperitoneal injection of cisplatin. Although the levels of renal GSH and Na^+^/K^+^-ATPase activity were decreased by cisplatin, nanocurcumin counteracted these effects, allowing renal cells to maintain the mitochondrial function [73]. While maintaining the polarized state of the plasma membrane could preserve the epithelial function of the renal tubercle, the inhibition of Na^+^/K^+^-ATPase in the absence of these nanoparticles caused extracellular fluid to leak into the kidney lumen [73]. Surprisingly, one study has revealed that curcumin nanoparticles enhance the expression of OCT2 in renal cells, which may be due to better solubility and physical–chemical properties of these nanoparticles than free curcumin [66]. Thus, other regulatory mechanisms can be involved here.

Also, the encapsulation of curcumin-cisplatin complex in mPEG-SS-PBAE-PLGA nanoparticles had nephroprotective effects on BalB/c mice. In these nanoparticles, polyethylene glycol (PEG) protected the complex against the reticuloendothelial system (RES), so the drug remained stable in the blood stream. However, the complex was released in the tumor microenvironment upon the pH turned acidic. It should be noted that the DNA binding ability of curcumin-cisplatin complex was similar to that of cisplatin. As a result, this complex could show more antimetastatic effects by inhibiting PI3K/AKT, matrix metallopeptidase 2 and vascular endothelial growth factor receptor 2 (VEGFR2) pathways, while it induced less renal effects by suppressing ROS production [74].

On the other hand, it has been reported that the nephroprotective effects of nanocurcumin is concentration-dependent so that 60 mg/kg body.weight (b.w) of nanoparticles could be more nephroprotective than 30 mg/kg b.w of them [72, 75]. Accordingly, in a clinical study of patients with localized muscle-invasive bladder cancer (MIBC), it was found that although well-tolerated nanocurcumin had no renal complication, patients did not respond well to the treatment. Thus, further studies are needed to determine the effective dose of nanocurcumin in the clinical studies [76].

### Liposomes

Liposome is an FDA-approved nanoparticle that are suitable for both targeted (adding ligands) and non-targeted (enhanced EPR effect) drug delivery methods [77].

In order to target cisplatin delivery and reduce its systemic cytotoxicity, anti-epidermal growth factor receptor (EGFR) antibody, which is against EGFR overexpressed on the surface of some cancer cells, can be attached to CDDP liposomal nanoparticles [78]. On the other hand, cisplatin-sodium alginate (SA) encapsulated in EGF-modified liposome, in which CDDP was encapsulated into the hydrophilic core of liposome, could also enhance the bioavailability and efficacy of CDDP delivery to the tumor and reduce kidney complications. Interestingly, while cisplatin has a low aqueous solubility, its binding to anionic SA can greatly increase its solubility [79].

Targeting tumors with nanoliposomes carrying both cisplatin and RNAs, such as miRNA and siRNA, can also be a nephroprotective approach, which works through changing tumor signaling pathways. RNA-based drug delivery systems have some problems, including rapid RES clearance and enzymatic degradation, short circulating lifetime, and higher cytotoxicity. However, formulating them with liposome can overcome these problems [80]. In this regard, multilayered layer by layer (LbL) nanoparticles (liposomes/poly-l-arginine (PLA)/siRNA of Kristen ras sarcoma viral oncogene homolog (siKRAS) and miR-34a/PLA/HA) containing CDDP were synthesized by encapsulating this drug in the hydrophilic core of phospholipid liposomes. The negatively charged hyaluronic acid was deposited as the last layer to extend blood circulation time and to target the overexpressed CD44 on lung adenocarcinoma cells, which reduce systemic toxicity of CDDP. The siKRAS and miR-34a can regulate KRAS oncogene and restore p53 function, respectively, which block the tumor defense pathways. Therefore, these nanoparticles reduced drug resistance. Moreover, LbL nanoparticles are promising due to their controllable size, high loading capacity, enhanced stability, staged cargo release, and their ability to surface modification [80].

Besides, liposomal cisplatin or lipoplatin are considered as the most effective nano-formulations of cisplatin that have even reached phases 1, 2 and 3 trials [81]. In one study, both monotherapy and combined treatment of lipoplatin with gemcitabine, 5-fluorouracil-leucovorin and paclitaxel were able to keep creatinine at a normal level in patients with one of lung, bladder and gastrointestinal cancers, together with a kidney disease [81]. In another study, cisplatin conjugated to 1-palmitoyl-2-glutaryl-sn-glycero-3-phosphocholine was assembled in the bilayer of liposomal cisplatin composed of 1,2-distearoyl-sn-glycero-3-phosphoethanolamine (DSPE), 1,2-distearoyl-sn-glycero-3-phosphoethanolamine-N-[Methoxyl(polyethyleneglycol)2000] (MPGG2k-DSPE and cholesterol to show a sustainable release of drug. By increasing the size of cisplatin in this way, these nanoparticles reduce the renal clearance of cisplatin, resulting in less nephrotoxic effects [82].

To develop a formulation with an enhanced liposomal loading capacity for curcumin, an automated microfluidic technology was applied. These liposome-curcumin nanoparticles containing cisplatin could reduce the dose-limiting effects of cisplatin on renal cells after a single dose injection to Balb/c mice [83]. Honokiol liposomes, which are composed of liposome and Honokiol, a lipophilic polyphenolic compound derived from *Magnolia officinalis*, can preserve the redox state of the cell and inhibit the apoptosis caused by caspase-3 in kidney cells. Therefore, the intravenous tail vein injection of these nanoparticles into mice was able to reverse AKI by inhibiting inflammation and fibrosis [84].

To reduce the systemic effects of cisplatin through controlling drug release, a microneedle technique was used to deliver cisplatin-loaded nanoliposomes via skin to head and neck tumors of xenograft mice. These nanoparticles (LCC-NPs) were synthesized by 1,2-dioleoyl-3-trimethy-lammoniumpropane (DOTAP), 1,2-dioleoyl-sn-glycerol-3-phospate (DOPA), cholesterol and DSPE-PEG-ammonium salt. DOPA-encapsulated CDDP not only could prevent aggregation and control liposome size, but also could be dispersed in water to prepare a coating bilayer through adding other mentioned lipids. Moreover, DOTAP allowed liposome to escape from the endosomes and accumulate less in the lysosomes; thus, the degradation of liposome in cytoplasm could induce CDDP release, resulting in the cell cycle arrest at G1 phase and apoptosis. Cholesterol, however, improved selectivity of liposome to tumor. In addition, DSPE-PEG-AA helped these nanoparticles target tumors by attaching its anisamide moiety to the overexpressed sigma receptors on human tumors. These nanoparticles were also pH sensitive and could release cisplatin only into the acidic environment of the tumor, thus reducing systemic toxicity of cisplatin [85].

### Chitosan (CS)

These biocompatible and biodegradable nanoparticles have some specific features like, high drug loading capacity, cell membrane permeability, pH-dependent therapeutic unloading and long circulation time [86]. The reactive amino group of glucosamine in the C2 position of chitosan is used to binding with drugs and targeting moieties of cells [87]. Hence, chitosan derivatives can reduce nephrotoxicity of cisplatin. Among the chitosan-based nanocarriers, such as N-naphthyl-N,O-succinyl chitosan (NSCT), N-benzyl-N,O-succinyl chitosan (BSCT) and N-octyl-N-O-succinyl chitosan (OSCT), BSCT nanoparticles displayed the highest loading efficiency (LE) of cisplatin, which can be attributed to the higher degree of succinic group on the polymer chain of BSCT compared to the NSCT and OSCT polymer chains [88].

Chitosan polymers can interact with cisplatin through co-ordinate bonds, which attach the carboxylic group of the polymer to the Pt of cisplatin at pH 8.5, so these bonds may be broken at the acidic pH of tumor. In this way, cisplatin will gradually be released into tumor. Therefore, with the targeted release of cisplatin near the tumor tissue, the effects of systemic toxicity such as acute and chronic nephrotoxicity can be eliminated [23, 88]. The treatment of normal renal cells, i.e., RPTEC/TERT1, with cisplatin-loaded nanocarriers showed a lower percentage of necrotic or late apoptotic cells compared with the cells treated with free cisplatin in one study, which might be due to the gradual release of cisplatin from the nanocarriers. In cancer cell lines, while nanocarriers showed a larger percentage of early apoptosis, free cisplatin presented a higher percentage of late apoptosis and necrosis. Early apoptosis is a process that takes place without inflammation when the membranes are intact. Once apoptotic cells lost their membrane integrity, late apoptosis and necrosis can be triggered, resulting in inflammatory responses. Therefore, nanocarriers could induce less inflammatory response in cancer cells compared to free cisplatin [88].

Another formulation of cisplatin complexed with γ-polyglutamic acid (γ-PGA) and CS has also showed fewer toxic effects on renal cells of mice. The γ-PGA/CDDP-CS complex exhibited a pH-dependent release of cisplatin in an in-vitro study after 12 days. At pH 7.4, less cisplatin was released from the γ-PGA/CDDP-CS complex [89]. This could be due to the electrical interaction of CS with the complex, while at pH 5.5 the release of cisplatin was higher [89, 90]. More importantly, despite a high concentration of CDDP in the complex, histological evidence did not show any kidney damage. Indeed, after intraperitoneal administration of the γ-PGA/CDDP-CS particles, most of them were trapped by RES and remained there before being released into the bloodstream. Subsequently, γ-PGA/CDDP-CS particles gradually released from RES, accumulated in the tumor tissue, and released cisplatin in a pH-dependent manner. Therefore, γ-PGA/CDDP-CS formulation is suitable for effective antitumor therapy and reduction of cisplatin-induced nephrotoxicity [89].

Moreover, irradiated chitosan-coated cisplatin and MgO nanoparticles (CHIT/Cis/MgO NPs), named cisplatin nanocomposite (Cis NC), apply minimal stimulatory effects on renal apoptotic and inflammatory cascades, so they are formulated to improve therapeutic efficacy while reducing nephrotoxicity. Anees et al. have shown that the oxidative stress, inflammation and apoptosis were reduced in renal cells of male Wistar rats treated with cisplatin nanocomposite through the regulation of AMPK/PI3K/Akt-mTOR and signal transducer and activator of transcription 1 (STAT1)/p53 signaling pathways [87]. Although cisplatin can activate STAT1 via the induction of ROS and NADPH oxidase activity, and then trigger inflammatory effectors (e.g., iNOS, TNF-α and IL-1β) and apoptotic factors (e.g., p53 and caspases), Cis NC did not significantly induce such changes in the kidneys of treated mice [87]. Also, self-assembly hybrid nanocomposites of CDDP-chitosan have been introduced as promising nano-drug with less nephrotoxicity, as shown in Table 3 [91].

Besides, a study used chitosan/siRNA nanoparticles to passively target kidneys. These nanoparticles altered CDDP-induced apoptotic proteins, e.g., p53, protein kinase C-δ (PKCδ) and GGT, by gene therapy to protect kidneys against CDDP-induced proximal tubule damages [92].

### Poly (lactic-co-glycolic acid) (PLGA)

These nanoparticles have numerous advantages like biocompatibility, biodegradability, high drug loading capacity, sustained release, and high permeability [93]. PLGA-based drug delivery systems can decrease systemic toxicity of cisplatin using a more selective and controllable approach [94]. Cisplatin is insoluble in organic solvents and partly soluble in water; hence, the external aqueous phase of these nanoparticles is saturated with cisplatin in order to elevate their drug loading profile [95]. The release pattern of cisplatin from the nanoparticles occurs in two phases. An initial burst release of drug can be attributed to the cisplatin present near the surface, and subsequently, a sustained release pattern will be occurred in response to the degradation of the polymer and releasing cisplatin from the nanoparticle matrix [96]. After treating mice with cisplatin loaded PLGA nanoparticles, their histological examination did not show any kidney damage [96].

In one study, the combinational therapy of PLGA nanoparticles with Boldine (Bol), which is an antioxidant compound with the ability to reduce cisplatin-induced nephrotoxicity, was investigated on swiss albino mice [97]. PLGA-encapsulated nano-Boldin (NBol) could reduce the cisplatin-induced nephrotoxicity by increasing SOD activity and decreasing LPO level. Furthermore, it was shown that NBol nanopolymers could enter into the cells faster than Bol and prevent DNA damage induced by cisplatin in normal cells [97]. Moreover, N, N’-diphenyl-1, 4-phenylenediamine loaded PLGA nanoparticles (Nano-DPPD) showed an anti-fibrotic activity against CDDP through decreasing CDDP-induced collagen contents in kidneys. These nanoparticles could also reduce CDDP-induced macrophages infiltration, tubular injury score and hydroxyproline contents, as a marker of DNA damage of renal cells [98]. The previous studies had shown that the encapsulation of thymoquinone (THY), as a potent antioxidant and anti-inflammatory compound, into PLGA and polyvinyl alcohol (PVA) polymers, and then using pluronic 127, as a non-anionic surfactant, could improve the poor solubility of THY and increase its bioavailability [99]. In this regard, the co-treatment of PLGA nanoparticle encapsulating THY (NP THY) with cisplatin can reduce cisplatin-induced nephrotoxicity in Ehrlich solid carcinoma (ESC) mice model without losing antitumor properties of cisplatin [100]. Kidney damage markers (i.e., uric acid, urea, creatinine, and cystatin c) were decreased in the treated mice in comparison with the cisplatin group. Also, NP THY protected against the oxidative stress caused by cisplatin via reducing the MDA of kidney tissue, increasing the rates of antioxidant markers (i.e., GSH, SOD, and CAT), and decreasing inflammatory marker levels (i.e., TNF-α, IL-1β, and NF-κB) (93).

### Micelles

CDDP-loaded polymeric micelles (CDDP-PMs) are more effective than cisplatin. In comparison with free cisplatin, CDDP-PMs can accumulate better in tumor, decrease renal exposure, and prolong blood circulation [101, 102]. The CDDP-PMs with mean size of 110 nm, loading capacity of 30% W/W, and ζ potential of -12 mV have lower drug-release rate in systemic circulation, but higher drug-release rate in tumor microenvironment [103]. In one study, while free cisplatin could significantly enhance BUN after 13 days, this marker remained at normal range in the mice treated with cisplatin/cl-micelles, even up to 28 days. Moreover, no histopathological alterations were detected in both bone marrow and kidney of the cisplatin/cl-micelle-treated group [101]. Polymeric micelles can have a hydrophilic shell made of PEG which helps these nanoparticles evade RES. By increasing the drug/copolymer ratios from 1:1 to 1:6, both nephrotoxicity and tumor inhibition rate of cisplatin decreases. However, CDDP-PMs with the ratio of 1:3 have the least toxicity and highest therapeutic effect. Also, cisplatin polymeric micelles can be designed to be pH-dependent and release cisplatin over than 10 days [102].

On the other hand, quercetin is an antioxidant flavonoid which can prevent nephrotoxicity and renal damage induced by cisplatin, methotrexate, ciprofloxacin, NaF, HgCl_2_ and cadmium [103]. However, quercetin is sensitive to temperature, hydroxylation, pH, metal ions and enzymatic activity [104, 105]. Therefore, in order to increase quercetin biological efficacy and bioavailability, some new formulations based on liposomes, nanoemulsions, nanoparticles and micelles have been synthesized [104]. Pluronic F127-encapsulated quercetin (P-quercetin) is a micellar formulation which has shown nephroprotective features at its lower concentrations [88, 106]. Also, quercetin and P-quercetin treatments identically decreased the tubular necrosis caused by cisplatin in cortical areas [105]. Besides, the chitosan polymeric micelles, including N-Octyl-Sulfate chitosan, N-Phthaloyl chitosan-g-mPEG, N-lauryl-carboxymethyl chitosan, and OSCS can also mitigate cisplatin-induced nephrotoxicity. It was identified that these chitosan derivatives have more water solubility and anticancer effects than chitosan, and can decrease the cisplatin-induced cytotoxicity in renal proximal tubular cells. Moreover, cisplatin-OSCS did not change the RPTEC/TERT cells viability [107]. In addition, Soodvilai et al*.* showed that the pre-treated with the Silymarin (SM)-loaded PMs could increase anticancer effects of cisplatin, while it decreased the necrosis and apoptosis in renal cells [108]. Also, the pre-treatment of cells with benzyl-functionalized succinyl chitosan (BSC) showed a renoprotective effect against cisplatin and other nephrotoxic drugs [109]. Furthermore, SM-loaded BSC PMs could improve the therapeutic effect and bioavailability of SM, and protect kidney against cisplatin [110]. However, both polymer types and the concentration of the SM incorporated in the PMs can change the cytotoxic effects of SM-PMs on RPTEC/TERT1 cells [108].

### Exosomes

Exosomes are 30–200 nm extracellular vesicles which have some specific CD-markers, like CD63 and CD9, and contain miRNAs, mRNAs, lipids and proteins [111]. It has been established that exosomes, especially those derived from human umbilical cord derived mesenchymal stem cells (HUMSC), can play a paramount role in the diagnosis and treatment of diseases. In this regard, we can take advantages of HUMSC exosomes to treat kidney disorders such as cisplatin-induced renal damage. In fact, the protective effects of HUMSC on the cisplatin-induced damages are present through inhibiting Bcl2 and increasing Bim, Bid, Bax, cleaved caspase-9, and cleaved caspase-3. In addition, HUMSC-exosomes have been able to rise the renal cell viability and the proportion of G1 phase cells. Moreover, the exosomes could inhibit cisplatin-caused apoptosis [112]. Moreover, a carbon monoxide(CO)-loaded hemoglobin-vesicle (CO-HbV) as renoprotectant has been able to reduce nephrotoxic effect of CDDP through inhibiting caspase-3-mediated apoptosis. CO delivery to tumor can contribute to tumor growth inhibition in B16-F10 melanoma cell-bearing mice because CO is toxic to tumor, but not normal tissues. This is due to the fact that binding CO to cytochrome c oxidase can trigger different responses in normal and cancer cells [113].

### Microspheres

Microspheres are spherical particles with the size ranging from 1 to 1000 μm. Gelatin microspheres (GM) can reduce nephrotoxic effects of cisplatin through offering a targeting drug delivery system. Cisplatin can enter into the hydrogel via a simple method, and then be released into tumor through the degeneration of the hydrogel. On the other hand, it has been established that cancer cells mostly secrete matrix metallopeptidase enzymes, like gelatinase and collagenase, that can be effective in the releasing CDDP near the tumor site by degradation of GMs [114].

### pH-sensitive polymeric nano-formulation

Lipid-coated cisplatin/oleanolic acid calcium carbonate nanoparticles (CDDP/OA-LCC NPs) are pH sensitive nanoparticles that show an increased tumor efficacy and blood circulation time [115]. Oleanolic acid (OA) is a pentacyclic triterpenoid which has both anti-oxidative and anti-inflammatory effects. The synergic effect of OA with CDDP is suitable for drug co-delivery. These nanoparticles have been able to decrease cisplatin-induced nephrotoxicity. In this regard, Shi et al. showed that OA could induce the antioxidant enzymes by activating the nuclear factor erythroid 2–related factor 2 (Nrf-2) [115]. In this way, it could eliminate the toxic effects caused by ROS. Moreover, NF-κB and AKT/mTOR pathways were deactivated by activating AMPK to reduce the release of pro-inflammatory cytokines and resistance to cisplatin [115]. Moreover, in another study a nanoparticle was designed using pH-sensitive CaCO3 cores (CDDP/OA-LCC NPs) to co-deliver CDDP and OA. Thus, Pt could release more at acidic condition. This is because the CaCO3 cores are stable at pH 7.4, while they rapidly collapse at pH 5.5 and release encapsulated drugs. This nano-drug not only showed better pharmacokinetic characteristics, e.g., prolonged blood circulation, selective tumor targeting, and higher antitumor efficacy, but also could mitigate CDDP-induced nephrotoxicity [115].

Another pH-sensitive nanoparticle is polyphosphazene-cisplatin (polycisplatin) that has been able to reduce the cisplatin nephrotoxicity by increasing drug accumulation in tumors. Although polycisplatin at the dose of 1.95 mg Pt/kg had less tumor suppressive effect than CDDP, it showed a better efficacy than CDDP at higher doses (> 3.9 mg Pt/kg). On the other hand, novel Pt-bisphosphonate polymer-metal complex nanoparticles (Pt-bp-NPs) are another pH sensitive nanoparticles that have led to a fast drug release in acidic extracellular environment of tumor, resulting in less systemic toxicity of cisplatin [116]. Cisplatin-loaded poly (l-glutamic acid)-g-methoxy poly ethylene glycol 5 k nanoparticles (PLG-g-mPEG 5 k) are also a pH and temperature sensitive nanoparticle which demonstrate longer blood circulation and reduced Pt accumulation in kidney, so they can decrease cisplatin-caused renal damages [117].

### Other nanoparticles

*-LHRH-peptide conjugated dextran nanoparticles:* gonadotropin-releasing hormone (GnRH) or luteinizing hormone-releasing hormone (LHRH) is a hormone that regulates the pituitary–gonadal axis. To create a targeting delivering system, this hormone is a suitable ligand because its receptor is overexpressed in most tumors. Dextran-SA-CDDP-LHRH has been able to increase the blood circulation of cisplatin and reduce the systemic toxicity and renal accumulation of this drug. Interestingly, while CDDP mostly was removed from kidneys, these nanoparticles were taken up by RES in mice [118].
*-Gallic acid-loaded eudragit-Rs 100 nanoparticles:* 3,4,5-trihydroxy benzoic acid (Gallic acid) has some polyphenolic compounds; hence, gallic acid and its nanoparticles have antioxidant, anti-inflammatory, antimutagenic, anti-carcinogenic, and mucoadhesive features. According to a previous study, both 10 mg/kg nano gallic acid and 50–100 mg/kg gallic acid can ameliorate the mitochondrial levels of MDA, ROS, TNF-α and IL-6 in renal, and increase the levels of mitochondrial antioxidant enzymes (e.g., SOD and CAT) [119].
*-Polymer nanosystems-Gambogic Acid-Urolithin A (P2Ns-GA-UA):* urolithin A (UA) is a metabolic compound with antioxidant and anti-inflammatory features that results from the transformation of ellagitannins by the gut bacteria. Compelling evidence indicated that oral administration of UA decreased the cisplatin-caused histopathological and morphological abnormalities, AKI and mortality in the rat models. This is because the UA nanoparticles can downregulate both p53-inducible genes and NRF2. Moreover, they maintain the levels of PARPΙ, AKI-related miRNA (miR-192-5p and miR-140-5p), intracellular NAD^+^, mitochondrial oxidative phosphorylation at their normal ranges. Furthermore, renal expression of NFR2-inducible genes [thioredoxin reductase I (Txnrd I), metallothionein (Mt I), sulfiredoxin I homolog (srxn I)], NFR2 protein, p53 protein and its inducible genes [i.e., activating transcription factor 3(Atf3), cyclin-dependent kinase inhibitor 1A (cdknla)(p21), transformation-related protein 53-inducible nuclear protein 1 (Trp53inp1)(sIp)] were significantly less in the mice treated with P2Ns-GA-UA compared to the cisplatin group. On the other hand, P2Ns-GA UA did not affect the hypoxic state of the renal. In general, P2Ns-GA UA treated group had less oxidative phosphorylation deficiencies and kidney apoptosis [120].
*-Cisplatin-polyacrylic acid (PAA) nano capsule (CDDP-PAA-NC):* conjugating CDDP to PAA helps to enhance drug loading and reduce drug leakage through replacing anionic chlorides in the drug with carboxylic residues in PAA. We can also inhibit the drug leakage via encapsulating this complex in PVA/superparamagnetic iron oxide (SPIO) shell. A prolonged blood circulation enhances the opportunity for nano-drugs to accumulate in tumor by leaky sites in vessels. Thus, these nanoparticles could successfully decrease the cisplatin-induced nephrotoxicity, while they increased CDDP tumor accumulation, and prolonged drug release [121].
-Hyaluronan–cisplatin conjugate nanoparticles (HCNPs) entrapped in Eudragit S100-coated pectinate/alginate microbeads (PAMs) (HCNP-PAMs): enzyme- or pH-dependent systems can be used to deliver drugs to colon. Pectin is a natural polysaccharide which can inhibit metastasis through attaching to the galectin-3 receptor. The use of pectin alone as a microencapsulation matrix needs high concentrations of pectin in electrospray method, owing to its low carboxyl groups; therefore, alginate is mixed with pectin to solve this problem. However, this matrix quickly releases drugs under gastric condition, which can be solved by coating Eudragit on the surface of these microbeads. Moreover, hyaluronan not only can form stable complexes with CDDP, but also can actively target CD44 receptors on cells, and ameliorate the cisplatin-induced nephrotoxicity [122].
*-Cysteine Pt(IV) prodrug NPs:* as mentioned earlier, intracellular thiol-containing molecules can induce cisplatin resistance in tumors. However, these nanoparticles are made of poly(disulfide amide) polymers that can reverse the drug resistance by a GSH-scavenging process, which rapidly induced the release of Pt ions in a thiol-rich medium. Thus, they are suitable for increasing CDDP efficiency, as they can deliver the active Pt to cisplatin-resistant cells and deplete GSH concentration in the cells. In addition, these nanoparticles activate apoptotic pathways by increasing p53 and caspase-3, and decreasing Bcl2. Interestingly, they had less systemic toxicity, including nephrotoxicity [123].
*-Hyaluronic acid-cisplatin/polystyrene-polymetformin (HA-CDDP/PMET):* it is used for co-delivery of metformin (MET) and CDDP to lung cancer. In a study, this nanoparticle could enhance the survival rate of animals without inducing nephrotoxicity. This was due to the fact that this nanoparticle could accumulate in the kidneys less than cisplatin. On the other hand, apoptosis was upregulated in tumor cell by the synergistic effect of CDDP and MET in HA-CDDP/PMET NPs, resulting in the regulation of cleaved PARP protein [124].
*-Aptamers (Apt):* Apts have some significant features, including great tissue penetration, low toxicity, lack of immunogenicity, thermal stability, high surface modification and being non-reactive to negatively charged proteins of blood circulation. Indeed, Apts interact only with their receptors on surface of tumor cells, resulting in less cisplatin accumulation in kidney and less nephrotoxicity in comparison with free CDDP at the same doses [125].
*-Glycyrrhetinic acid (GA) Aliginate acid (ALG) Pt nanoparticles:* alginate is a biodegradable and non-toxic polysaccharide which operating as the shell and skeleton of nanoparticles. The presence of several carboxy groups in the structure of this polymer give it a negative surface charge. Therefore, GA-ALG@Pt NPs cannot interact with blood elements and show low systemic toxicity and long blood circulation [126].
-*Silk fibroin peptide/baicalein nanofibers (SFP/BA NFs):* this nano-formulation could enhance the CDDP uptake and localization to mitochondria in renal cells, which led to an inhibition of the cisplatin-induced ROS formation and mitochondrial membrane potential disruption. As a result, they successfully protected against cisplatin-induced AKI via improving antioxidant responses, e.g., SOD and GSH, and suppressing DNA damage and the cyclic GMP-AMP synthase-stimulator of interferon genes (cGAS-STING) pathway activation in kidney [127].
*-Folate grafted albumin nanoparticles:* folic acid receptors are over-expressed on the surface of various cancer cells, so this vitamin can be used for targeted therapy. Based on one study, the administration of cisplatin loaded folic acid decorated bovine serum albumin nanoparticles (Cp-FA-BSA-Nps) limits the cisplatin adverse effects on kidneys [128].

### The histopathological effects of cisplatin and nanoparticles on kidneys

As shown in Table 3, both metallic and polymer nanoparticles have been able to reduce or completely remove the negative effects of cisplatin on kidney tissue in in-vivo studies. In this regard, the treatment of animals with cisplatin has been able to induce tubular, glomerular and interstitial damages. Among CDDP-induced tubular injuries, we can mention necrosis, atrophy, increase in eosinophilic material, dilation, vacuolation, cystic dilatation, cast formation, brush border damage, fibrosis, edema, hemorrhage, swelling, infiltration of inflammatory cells. Moreover, CDDP-induced glomerular damage includes congestion, Bowman's capsule collapse, atrophy, necrosis, thickening basement membrane, widening Bowman's space, and capsule deformation. As summarized in Table 3, we can see that the reviewed nanoparticles have been able to neutralize the histopathological effects of CDDP on renal tissues [54, 57, 58, 72, 87, 99, 120].

## Conclusion

This study was conducted with the aim of finding effective drug delivery systems to reduce the nephrotoxic effect of cisplatin. In this regard, nanomedicines based on metallic and polymeric nanoparticles were investigated between 2011 and 2022. The current review demonstrated that after treating with cisplatin, these nanoparticles can strengthen the renal antioxidant system and reduce ROS generation either by their innate antioxidant properties, such as cerium oxide, or by carrying antioxidants. In addition, cisplatin can activate various apoptotic and inflammatory pathways in kidneys, whereas both metallic and polymeric nanoparticles have been able to preserve the kidney tissue by regulating these pathways and maintaining the survival of renal cells. In fact, the present review shows that some cisplatin-based nanosystems with their renoprotective mechanisms are probably helpful in finding renoprotective formulations of cisplatin. Nanocurcumin could inhibit oxidative stress and acting as a ROS scavenger. CONPs could reduce lipid peroxidation and pro-inflammatory cytokines, such as IL-6 and TNF-α, and increase antioxidants, such as CAT and GSH, in cisplatin-treated mice. CDDP-loaded AgNPs could inhibit mitochondria-mediated apoptosis and the release of AIF and cytochrome c from mitochondria. In addition, Se@TE NPs could mitigate the increased level of dephosphorylated AKT, phosphorylated p38 MAPK and phosphorylated c-Jun N-terminal kinase (JNK) induced by cisplatin. Furthermore, PLGA-encapsulated nano-Boldin reduced the cisplatin-induced nephrotoxicity by increasing SOD activity and decreasing LPO level. Also, the co-treatment of PLGA nanoparticle encapsulating THY with cisplatin decreased cisplatin-induced nephrotoxicity in ESC mice model without losing antitumor properties of cisplatin as well as kidney damage markers were reduced in the treated mice in comparison with the cisplatin group. Moreover, exosomes as new nanoparticles could mitigate cisplatin-induced renal damage through inhibiting Bcl2 and increasing Bim, Bid, Bax, cleaved caspase-9, and cleaved caspase-3.

**Fig. 5:**
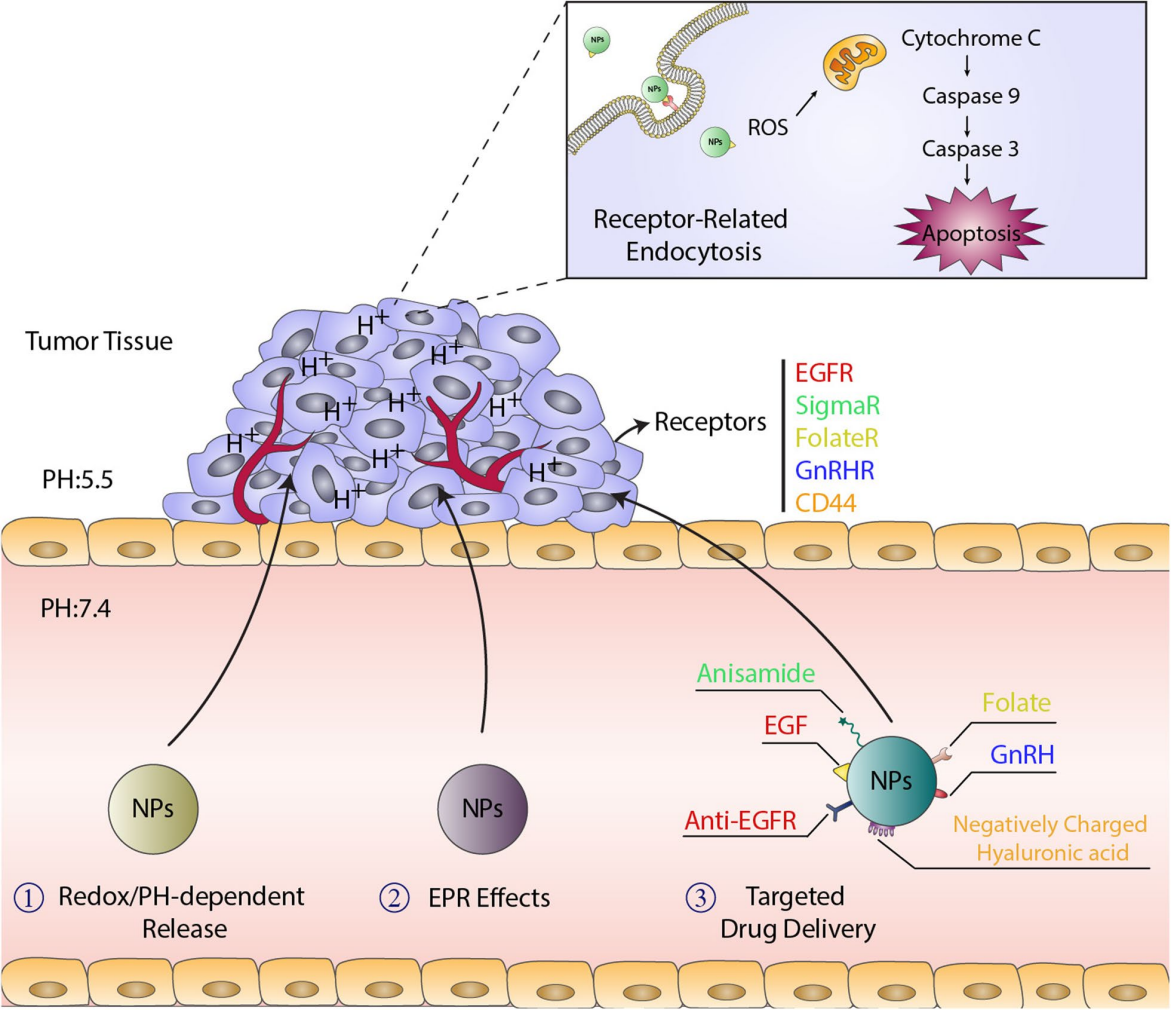
Improved anti-tumor activity of CDDP using the reviwed nanoparticles. The reviwed nanoparticles are able to mitigate CDDP-induced nephrotoxicity by improving targeted drug delivery using mechanisms like (1) redox/pH-dependent releases, (2) EPR effects, and (3) ligands used in NTDDS. Based on redox/pH-dependent release of CDDP, nanocarriers can release the drug in response to the difference between the extra- and intracellular pH or redox environments. In NTDDS approach, ligands on the surface of nanocarriers can bind to their overexpressed, specific receptors on the cell membrane of tumor cells, resulting in a receptor-related endocytosis and apoptosis. Enhanced permeability and retention (EPR); Epidermal growth factor (EGF); Gonadotropin-releasing hormone (GnRH); Reactive oxygen species (ROS); Nanoparticle-Mediated Targeted Drug Delivery System (NTDDS)

The reviewed renoprotective nanoparticles have been classified and summarized in Fig. 1, and their renoprotective mechanisms are shown in Figs. 2 and 3, and Tables 1 and 3. In addition, some of the reviewed nanoparticles not only mitigate CDDP-induced nephrotoxicity, but also improve the anti-tumor activity, loading efficiency, releasing efficiency, biodistribution, and biosafety of CDDP as summarized in Table 2. These nanoparticles can accumulate more in tumor sites and demonstrate more anti-tumor activity using some mechanisms like (1) redox/pH-dependent releases, (2) EPR effects, and (3) ligands used in nanoparticle-mediated targeted drug delivery system (NTDDS) (as shown in Fig. 5). Due to their renoprotective mechanisms and more anti-tumor activity, researchers must be aware in their attention to the nanoparticles, take correct steps for synthesis, loading efficiency, releasing efficiency, biodistribution, and biosafety. Nevertheless, more research is required to have a clear picture on the renoprotective mechanisms related to the nanoparticles in particular in clinical trial studies.

## Data Availability

Not applicable.

## Abbreviations

CDDP: Cisplatin
GSH: Glutathione
ER: Endoplasmic reticulum
CTR1: Copper transporter receptor 1
MATE1: Multidrug extrusion transporter-1
GST: Glutathione S-transferases
GGT: γ-Glutamyl transferase
PTECs: Proximal tubular epithelial cells
PARP1: Poly (ADP-ribose) polymerase-1
AIF: Apoptosis-inducing factor
TNFR: Tumor necrosis factor receptor
FasR: Fas receptor
CAT: Catalase
ROS: Reactive oxygen species
ATP: Adenosine triphosphate
NF-κB: Nuclear factor kappa B
MAPK: Mitogen-activated protein kinase
TNF-α: Tumor necrosis factor α
ICAM1: Intercellular adhesion molecule 1
CONPs: Cerium oxide nanoparticles
TAO: Total antioxidant
NP: Nanoparticle
iNOS: Inducible nitric oxide synthase
HO-1: Hemeoxygenase-1
AKI: Acute kidney injury
SeNPs: Selenium nanoparticles
JNK: C-Jun N-terminal kinase
Se-Met: Selenomethionine
Se­Cys: Selenocysteine
GSH-Px: Glutathione peroxidase
Sirt1: Sirtuin 1
SOD: Superoxide dismutase
NQO1: NAD(P)Quinine oxidoreductase1
NO: Nitric oxide
PEG: Polyethylene glycol
RES: Reticuloendothelial system
MMP: Mitochondrial membrane potential
VEGFR2: Vascular endothelial growth factor receptor 2
MIBC: Muscle-invasive bladder cancer
EGFR: Epidermal growth factor receptor
SA: Sodium alginate
DSPE: 1,2-Distearoyl-sn-glycero-3-phosphoethanolamine
NSCT: N-naphthyl-N,O-succinyl chitosan
BSCT: N-benzyl-N,O-succinyl chitosan
OSCT: N-octyl-N–O-succinyl chitosan
LE: Loading efficiency
γ-PGA: γ-Polyglutamic acid
STAT1: Signal transducer and activator of transcription 1
Bol: Boldine
NBol: Nano-Boldin
THY: Thymoquinone
PVA: Polyvinyl Alcohol
PLGA: Poly lactic co-glycolic acid
ESC: Ehrlich solid carcinoma
CDDP-PMs: CDDP-loaded polymeric micelles
OSCS: O-succinyl chitosan
SM: Silymarin
HUMSC: Human umbilical cord derived mesenchymal stem cells
CO: Carbon monoxide
CO-HbV: Carbon monoxide-loaded hemoglobin-vesicle
GM: Gelatin microspheres
CDDP/OA-LCC NPs: Lipid-coated cisplatin/oleanolic acid calcium carbonate nanoparticles
OA: Oleanolic acid
Nrf-2: Nuclear factor erythroid 2–related factor 2
polycisplatin: Polyphosphazene-cisplatin
Pt-bp-NPs: Platinum-bisphosphonate polymer-metal complex nanoparticles
GnRH: Gonadotropin-releasing hormone
LHRH: Luteinizing hormone-releasing hormone
Cp-FA-BSA-Nps: Cisplatin loaded folic acid decorated bovine serum albumin nanoparticles
P2Ns-GA-UA: Polymer nanosystems-Gambogic Acid-Urolithin A
UA: Urolithin A
Txnrd I: Thioredoxin reductase I
Mt I: Metallothionein
srxn I: Sulfiredoxin I
Atf3: Activating transcription factor 3
cdknla: Cyclin-dependent kinase inhibitor 1A
Trp53inp1: Transformation-related protein 53-inducible nuclear protein 1
HCNPs: Hyluronan-cisplatin conjugate nanoparticles
CP5: Cysteine prodrug 5
HA-CDDP/PMET: Hyaluronic acid-cisplatin/polystyrene-polymetformin
MET: Metformin
Apt: Aptamer
GA: Glycyrrhetinic acid
ALG: Aliginate acid
Pt: Platinum
EPR: Enhanced permeability and retention
OCT: Organic cation transporter
DOTAP: 1,2-Dioleoyl-3-trimethy-lammoniumpropane
DOPA: 1,2-Dioleoyl-sn-glycerol-3-phospate
PVA: Polyvinyl alcohol
HCNPs: Hyaluronan–cisplatin conjugate nanoparticles
PAMs: Pectinate/alginate microbeads
PLA: Poly-L-arginine
PKCδ: Protein kinase C-δ
PAA: Polyacrylic acid
SFP: Silk fibroin peptide
BA NFs: Baicalein nanofibers
cGAS-STING: Cyclic GMP-AMP synthase-stimulator of interferon genes
MDA: Malondialdehyde
TP: Tea Polyphenols
KRAS: Kristen ras sarcoma viral oncogene homolog
LbL: Layer by layer
SPIO: Superparamagnetic Iron Oxide
NTDDS: Nanoparticle-Mediated Targeted Drug Delivery System
